# Linear Aminolipids with Moderate Antimicrobial Activity from the Antarctic Gram-Negative Bacterium *Aequorivita* sp.

**DOI:** 10.3390/md16060187

**Published:** 2018-05-28

**Authors:** Giuseppina Chianese, Fortunato Palma Esposito, Delphine Parrot, Colin Ingham, Donatella de Pascale, Deniz Tasdemir

**Affiliations:** 1 GEOMAR Centre for Marine Biotechnology (GEOMAR-Biotech), Research Unit Marine Natural Products Chemistry, GEOMAR Helmholtz Centre for Ocean Research Kiel, Am Kiel-Kanal 44, 24106 Kiel, Germany; g.chianese@unina.it (G.C.); dparrot@geomar.de (D.P.); 2 Institute of Protein Biochemistry, National Research Council, Via P. Castellino, 111, I-80131 Naples, Italy; f.palma@ibp.cnr.it (F.P.E.); d.depascale@ibp.cnr.it (D.d.P.); 3 Hoekmine BV, 3584 CS Utrecht, The Netherlands; colinutrecht@gmail.com; 4 Faculty of Mathematics and Natural Sciences, Kiel University, Christian-Albrechts-Platz 4, 24118 Kiel, Germany

**Keywords:** *Aequorivita*, Gram-negative bacterium, miniaturized culture chip, linear aminolipid, MRSA, LC-MS/MS

## Abstract

The combination of LC-MS/MS based metabolomics approach and anti-MRSA activity-guided fractionation scheme was applied on the Gram-negative bacterium *Aequorivita* sp. isolated from shallow Antarctic sea sediment using a miniaturized culture chip technique. This methodology afforded the isolation of three new (**1**–**3**) and four known (**4**–**7**) N-terminal glycine- or serine-bearing *iso*-fatty acid amides esterified with another *iso*-fatty acid through their C-3 hydroxy groups. The chemical structures of the new compounds were elucidated using a set of spectroscopic (NMR, [α]_D_ and FT-IR) and spectrometric (HRMS, HRMS/MS) methods. The aminolipids possessing an N-terminal glycine unit (**1**, **2**, **4**, **5**) showed moderate in vitro antimicrobial activity against MRSA (IC_50_ values 22–145 μg/mL). This is the first in-depth chemistry and biological activity study performed on the microbial genus *Aequorivita*.

## 1. Introduction

The diversity of microbes on earth is enormous. However, despite all the progress made in microbiology over more than 100 years, still only a little fraction (<1%) of microbial species can be grown in artificial media. The remaining 99% remain resistant to standard cultivation techniques, leading to the famous “dark matter” phenomenon in microbiology [1]. Cultivability is not only a main issue in microbial ecology studies, but also the major obstacle in exploration of bacteria for their bioactive constituents, e.g., for discovery of antibiotics. Several techniques, such as diffusion chambers [2] or the iChip [3] have been used to increase microbial cultivability. The iChip, a multichannel device consisting of hundreds of miniature diffusion chambers and semipermeable membranes increases the cultivability rate up to 50% [3] as the device is placed in the natural environment with all factors required for growth being present. The iChip has been used to simultaneously isolate and grow uncultured bacteria, including the soil *β*-proteobacterium *Eleftheria terrae*, which yielded teixobactin, a depsipeptide antibiotic inhibiting the cell wall synthesis without detectable resistance [4]. A similar approach is the use of a Miniaturized Culture Chip (MCC) [5,6], which is also directly placed on natural sediments. The nutrients and signaling molecules from the microbial community present in the sediment can diffuse into wells through a highly porous ceramic membrane (which acts as a sterile filter), thus simulates a natural environment. This allows microbes to grow in the microwells on the upper side of the chip as segregated microcolonies leading to the isolation of previously uncultivated microorganisms. The MCC has two differences with the iChip in design and use: (1) the cultivation wells are open allowing microscopy of the growing microcolonies and therefore selection is possible based around phenotype and (2) the large number of wells (4000 to hundreds of thousands) allow an inoculation strategy where only a fraction of the wells are occupied by a growing colony, which is likely to be a single strain protected from antagonism by neighbours [6]. 

Gram-negative bacteria are a very large and taxonomically diverse group of microorganisms with high survival and adaptation capability in every environment [7]. Although the current biodiscovery studies mostly focus on Gram-positive Actinobacteria, Gram-negative bacteria are being increasingly recognized as a prolific source of diverse molecules with wide ranging biological activities [7,8]. A prominent example of a molecule from this group of marine bacteria is didemnin B, the first marine natural product that entered clinical trials against cancer. This compound, originally reported from the Caribbean tunicate *Trididemnum solidum* was shown in 2011 to be produced by the Gram-negative *α*-proteobacterium *Tistrella mobillis* [9]. Another eminent example is represented by the bryostatins, complex polyketides that modulate protein kinase C. Bryostatins are potent anticancer and neurological agents that have been linked to an as yet uncultured Gram-negative *γ*-proteobacterial symbiont of the marine bryozoan *Bugula neritina*, *Candidatus Endobugula sertula*. A bryostatin polyketide synthase gene cluster has been identified from Ca. *E. sertula* [10]. Several recent reviews have highlighted the chemical diversity, bioactivity and biosynthetic machinery of the Gram-negative bacteria [7,11,12], pointing out their untapped potential in discovery of new, bioactive molecules. 

*Aequorivita* is a small genus of the Gram-negative bacterial family Flavobacteriaceae. This genus was first discovered in 2002 from Antarctic habitats [13]. The existing studies on the few known members of *Aequorivita* have only dealt with the taxonomy, fatty acid composition and DNA G+C content [13], but no in-depth chemical or bioactivity study has been performed on any member of this genus so far. By employing the MCC method, we have isolated from Antarctic shallow sea sediment an *Aequorivita* sp. that showed moderate in vitro activity against MRSA. The combination of the traditional (MRSA)-activity guided isolation scheme with LC-MS^2^-based metabolomics approach allowed the dereplication and subsequent isolation of aminolipids from the bioactive fractions of this bacterium. The further purification steps afforded three new (**1**–**3**) and four known (**4**–**7**) linear aminolipids that are glycine (Gly) or glycyl-serine (Gly-Ser)-bearing *iso*-fatty acid amides esterified with another *iso*-fatty acid through their C-3 hydroxy groups. Herein, we describe the details of LC-MS^2^-based metabolomics studies along with the isolation, structural elucidation and the antimicrobial activity of compounds **1**–**7**.

## 2. Results

**Figure 1:**
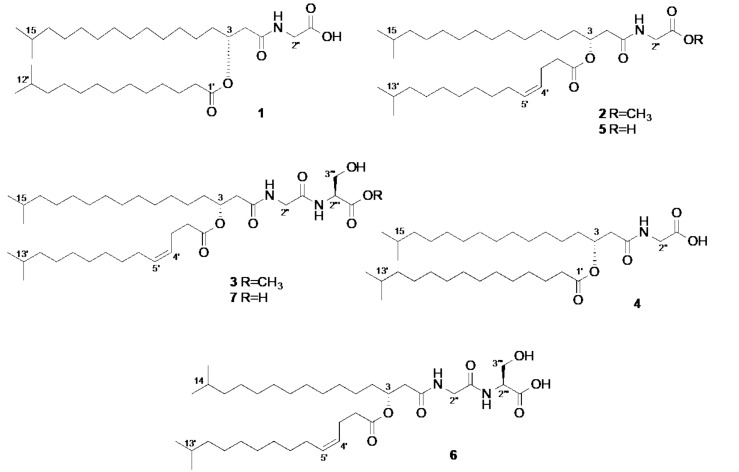
Chemical structures of the aminolipids (**1**–**7**) isolated from *Aequorivita* sp.

The EtOAc extract of the bioactive pellet (anti-MRSA IC_50_ value 120 µg/mL) was fractionated over a C18 SPE cartridge to yield six fractions. The activity was tracked to fractions 5 and 6, but due to little amounts of fraction 5, only fraction 6 was studied by LC-MS/MS-based metabolomics followed by purification studies. An in-depth manual dereplication of this fraction using multiple commercial and public databases led to the annotation of four known aminolipids, two with N-terminal glycine unit, i.e., compounds **4** (*m*/*z* [M + Na]^+^ 590.4759, C_34_H_65_NO_5_Na) and **5** (*m*/*z* [M + Na]^+^ 588.4617, C_34_H_63_NO_5_Na), and two with N-terminal serine moiety, WB-3559 A (**6**, *m*/*z* [M + Na]^+^ 661.4747, C_36_H_66_N_2_O_7_Na) and WB-3559 B (**7**, *m*/*z* [M + Na]^+^ 675.4936, C_37_H_68_N_2_O_7_Na) (Figure 1). The HRMS^2^ fragmentation pattern was most helpful in rapid identification of the chain length and unsaturation level of *iso*-fatty acyl moieties following the cleavage of the ester bond at C-3 (Figure S20). The HRMS/MS spectrum of **4** showed a characteristic fragment ion at *m*/*z* [M + Na]^+^ 348.2568, corresponding to the loss of 242 Da (C_15_H_30_O_2_). This led to the assignment of the (lower) fatty acyl moiety as *iso*-pentadecanoic acid in **4**. The HRMS^2^ spectra of the compounds **5**, **6** and **7** contained fragments due to loss of 240 Da ([M + Na−C_15_H_28_O_2_]^+^), indicating the presence of a double bond. Thus, the (lower) fatty acyl moiety was identified as *iso*-pentadecenoic acid in compounds **5**–**7**. The additional minor fragments observed at *m*/*z* 435.2878 in **6** and *m*/*z* 449.3053 in **7**, correspond to the loss of 226 Da ([M−C_7_H_11_N_2_O_5_Na]^+^), confirming the amino acid residue as glycyl-serine. The combination of those fragments permitted to determine, by deduction, the chain length of the upper fatty acid esterified at (up to) C-3 as C_14_ and C_15_ in compounds **6** and **7**, respectively. 

As LC-MS^2^ analyses could not annotate several compounds in multiple databases, we decided to undertake purification studies on the SPE fraction 6 to obtain potentially new metabolites. Repeated RP-HPLC separation of this fraction afforded a new aminolipid (**1**) and two new methyl ester derivatives (**2** and **3**), in addition to the four known metabolites described above, **4** [14,15,16], **5** [15], WB-35559 A (**6**) [17,18] and WB-35559 B (**7**) [17,18] (Figure 1). The structures of the known compounds were confirmed by comparison of their NMR, [α]_D_ and LC-HRMS and MS/MS data with those previously reported [14,15,16,17,18].

**Figure 2:**
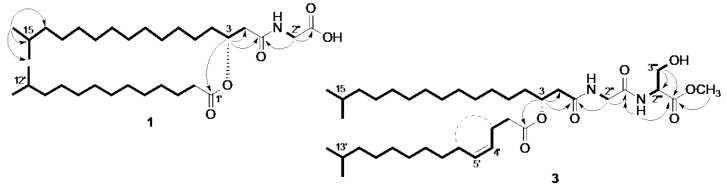
The COSY (in bold), key H→C HMBC (arrows) and H→H NOE (dashed line) correlations observed for the new compounds **1** and **3**.

The new compound **1** was isolated as yellow oil with the molecular formula C_33_H_63_NO_5_ as deduced by HRESIMS (*m*/*z* 576.4603, [M + Na]^+^). The ^1^H and ^13^C NMR data (Table 1 and Table 2) contained characteristic resonances of a linear N-terminal amino acid-bearing *iso*-fatty acid amide esterified with another *iso*-fatty acid, including a glycine residue (*δ*_C_ 171.1, C-1′′; *δ*_H_ 4.07, H_2_-2′′, *δ*_C_ 41.1 C-2′′), an oxymethine (*δ*_H_ 5.16, *δ*_C_ 71.1, H-3), an amide carbonyl at *δ*_C_ 170.5 (C-1) and an ester carbonyl at *δ*_C_ 173.7 (C-1′). Also evident were two sets of isopropyl termini (*δ*_H_ 0.86, *d*, *J* = 6.7 Hz, 12H, CH_3_-16, CH_3_-17, CH_3_-13′ and CH_3_-14′; 1.51, m, 2H, H-15 and H-12′) plus strongly overlapped methylene signals around *δ*_H_ 1.25 (m) characteristic of (an) aliphatic fatty acid chain. Two complex multiplets downshielded to *δ*_H_ 2.54 (CH_2_-2) and *δ*_H_ 2.30 (CH_2_-2′) were attributed to methylene groups adjacent to two carbonyl groups (C-1 and C-1′, respectively). The remaining signals were assigned with the aid of 2D NMR experiments. The COSY and the key HMBC correlations are depicted in Figure 2. Briefly, the presence of a glycine moiety was supported by the observed COSY correlations between the amide proton (Gly)NH at *δ*_H_ 6.38 and H_2_-2′′, as well as the HMBC correlations observed from H_2_-2′′ to both C-1′′ and C-1. The oxymethine H-3 scalarly coupled with two methylenes (*δ*_H_ 1.62, 2H, m, H-4; *δ*_H_ 2.54, 2H, m, H-2) in the COSY spectrum. The position C-3 was identified as the site of esterification with a (lower) *iso*-fatty acyl moiety based on the HMBC correlation of H-3 with the ester carbonyl at C-1′ plus the additional HMBC correlations, namely H_2_-2/C-3, H-3/C-1, H-3/C-2, H-3/C-4, H_2_-2′/C-1′, and H-3′/C-1′. The MS/MS fragment ion at *m*/*z* [M + Na]^+^ 348.2515 (C_19_H_35_NO_3_Na) corresponding to [M + Na−C_14_H_28_O_2_]^+^ allowed the identification of the (lower) *iso*-fatty acyl moiety as 12-methyl-tridecanoic acid (=*iso*-tetradecanoic acid). Compound **1** is structurally identical with compound **4**, with the exception of the alkyl chain length of the (lower) fatty acyl portion, which is *iso*-pentadecanoic acid in **4**. Due to the complexity of H-3 signal in the ^1^H NMR spectrum, a coupling constant (*J*) analysis was not possible, hence the stereochemical assignment of the C-3 was solely based on its [α]_D_ value. The positive sign of the specific rotation value (${\left[\alpha \right]}_{D}^{\mathrm{22}}$ + 45, CHCl_3_) and structural analogy to the known compound **4** (${\left[\alpha \right]}_{D}^{\mathrm{22}}$ + 0.77, CHCl_3_) [15], it is biosynthetically reasonable to assume that **1** has the same (*R*) configuration at C-3. Thus, the new compound **1** was identified as *R*-(+)-*N*-[15-methyl-3-(12-methyltridecanoyloxy)-hexadecanoyl]glycine.

The compound **2** (*m*/*z* [M + Na]^+^ 602.4767), also a yellow oil, was assigned the molecular formula of C_35_H_65_NO_5_ by HRESIMS. Based on the comparison of its NMR and HRESIMS/MS data with other purified compounds, **2** was identified as the methyl ester of the known compound **5**. The difference of 14 Da with the molecular formula of **5** (*m*/*z* [M + Na]^+^ 588.4617) indicated the presence of a methyl ester function in **2**. The detailed analysis of the 2D NMR spectra confirmed the full spin system of the (lower) *iso*-fatty acyl moiety and the position of the double bond between C-4′ and C-5′. The key COSY correlations were observed between the olefinic protons H-4′ (*δ*_H_ 5.30) and H-5′ (*δ*_H_ 5.40), as well as between H-4′ and H_2_-3′ (*δ*_H_ 2.34) and H-5′ and H_2_-6′ (*δ*_H_ 2.02). The diagnostic HMBC correlations were those between H_2_-3′/C-2′, H_2_-3′/C-1′ and H-5′/C-4′, H-5′/C-3′. The HMBC cross peak between the methoxyl signal (*δ*_H_ 3.75 s) and C-1′′ (*δ*_C_ 170.2) confirmed the methylation of the carboxylic acid of the N-terminal glycine residue (Figure 1). The complexity of the H-4′ and H-5′ refrained us doing coupling constant (*J*) analysis, but the clear NOE correlation between H_2_-3′ and H_2_-6′ indicated the *Z* configuration of the double bond Δ^4′^. The analysis of the MS/MS fragmentation pattern of **2** supported the assignment of the (lower) fatty acyl moiety, as in compounds **5**–**7**, as *iso*-pentadecenoic acid (fragment ion at *m*/*z* [M + Na]^+^ 362.2674 corresponding to a loss of 240 Da equivalent to C_15_H_28_O_2_). The stereochemical configuration of the C-3 atom was assigned as *R* based on the positive specific rotation value (${\left[\alpha \right]}_{D}^{\mathrm{22}}$ + 8.5, CHCl_3_), analogous to that of **5** (${\left[\alpha \right]}_{D}^{\mathrm{22}}$ + 0.45, CHCl_3_) [15]. Since MeOH was never used in the extraction or in the isolation procedure and the residual signal of the methoxyl group was visible in the ^1^H-NMR spectrum of the crude EtOAc extract, compound **2** cannot be considered an artifact. Thus, the chemical structure of the new compound **2** is *R*-(+)-*N*-[15-methyl-3-(13-methyl-4Z-tetradecenoyloxy)-hexadecanoyl]glycine methyl ester.

The molecular formula of compound **3** was established as C_38_H_70_N_2_O_7_ by HRESIMS (*m*/*z* [M + Na]^+^ 689.5085). Based on the comparison of the NMR and HRESIMS/MS data, **3** was identified as methyl ester of the known compound **7** ([M + Na]^+^ 675.4936, difference of 14 Da in their molecular formulae). The N-terminal amino acid residue was identified as serine based on the 2D COSY NMR spectrum that contained a short spin system including the amide proton (*δ*_H_ 6.90, 1H, *d*, (Ser)NH), the diastereotopic methylene H_2_-3′′′ (*δ*_H_ 3.96, 1H, H-3′′′a; *δ*_H_ 4.00, 1H, H-3′′′b) and the oxymethine H-2′′′ (*δ*_H_ 4.66, 1H, m). Figure 2 depicts the COSY as well as the key HMBC and NOESY correlations observed for compound **3**. Briefly key HMBC correlations observed for compound **3** include (Ser)NH/C-1′′′, OCH_3_/C-1′′′, H-2′′′/C-1′′’ and H_2_-3′′′/C-1′′′. The configuration of the Δ^4′^ was assigned as *Z* based on the NOE correlation between H_2_-3′/H_2_-6′ (Figure 2). Similar to **7**, the MS/MS spectrum of **3** revealed a fragment at *m*/*z* [M + Na]^+^ 449.2977 due to loss of 240 Da (C_15_H_28_O_2_) confirming the assignment of the (lower) fatty acyl moiety as *iso*-pentadecenoic acid. Compound **3** has previously been synthetized by Uchida et al. (1985) starting from the aminolipid named (3*R*, l-serine)-WB-3559B [18], but it has never been reported as a natural product before. Based on (i) the strict biosynthetic relationship to the known compound **7** (ii) the fact that all serine residues in WB3559 metabolites [17,18] have been reported with L-configuration, (iii) the positive optical rotation value (${\left[\alpha \right]}_{D}^{\mathrm{22}}$ + 45, CHCl_3_) comparable to that of **7** (${\left[\alpha \right]}_{D}^{\mathrm{22}}$ + 16.7, CHCl_3_) [18], it is reasonable to assume that **3** has the same (*R)* configuration at C-3 and contains *L*-serine as N-terminal amino acid. Again, as we never used MeOH in the extraction or in the isolation procedure, compound **3** should be considered a genuine natural compound, and no artifact. Thus, the new compound **3** is *N*-[*N*-[3(R)-15-methyl-3-(13-methyl-4Z-tetradecenoyloxy)-hexadecanoyl]glycyl]-l-serine methyl ester.

Compounds **1**–**7** were tested in vitro for their antimicrobial activity against MRSA. As shown in Table 3, all four N-terminal glycine-containing aminolipids (**1**, **2**, **4**, **5**) exhibited higher to equal activity to the crude EtOAc extract (IC_50_ value 120 µg/mL). The IC_50_ values observed for each compound were as follows: **1** (58 µg/mL), **2** (145 µg/mL), **4** (22 µg/mL) and **5** (93 µg/mL). These results indicate that the methylation of the N-terminal amino acid residue or the introduction of a double bond in the lower *iso*-fatty acyl chain diminishes or totally abolishes the antibiotic activity (IC_50_ > 200 µg/mL). In addition, all N-terminal serine-bearing analogues (**3**, **6**, **7**) were devoid of any inhibitory potential even at the highest test concentrations (200 µg/mL), suggesting that the glycine is the favoured N-terminal amino acid. 

## 3. Discussion

Back in 1999, Batrakov et al. suggested the phosphorus-free lipoaminoacids to be widely distributed among Gram-negative bacteria [16]. Indeed, the Gram-negative bacteria have frequently been reported as producers of aminolipids [19,20,21] detected in complex total lipid fractions. However, due to difficulties in their purification, they were mostly left as “unidentified aminolipids” without further isolation or chemical characterization [22,23]. Only a few *N*-(*β*-acyloxyacyl) amino acids have been reported from the Gram-negative order Flavobacteriales including the members of the families Flavobacteriaceae (e.g., *Flavobacterium* sp.) and Cytophagaceae (e.g., *Cytophaga* and *Cyclobacterium* sp.) [14,15,16,17,18]. They are often lipomonopeptides or lipodipeptides containing glycine, serine, ornithine or glycyl-serine as amino acid residue and an *iso*-fatty acid ester at C-3 with different degrees of unsaturation [15,16,18,24]. Ornithine and serine-containing (amino)lipids of some Gram-negative pathogenic bacteria are known to hemagglutinate human and rabbit erythrocytes [25]. Fibrinolytic [17], macrophage activation [26], *N*-type calcium channel blockage [15] and biosurfactant type activities [24,27] have also been reported for this chemical family. While some natural cyclic aminolipids (short lipopeptides) have been shown to exert antibacterial effects [28], the only linear aminolipids with demonstrated antibacterial activity are the synthetically prepared molecules, e.g., *N*-stearoyl proline [29]. Hence, it appears that this is the first study reporting the antimicrobial activity of linear aminolipids of bacterial origin. Odd-numbered fatty acids are frequent in bacteria with C15:0, C17:0 or C19 lipids being the most common ones [30]. The isolation of mixed odd and even *iso*-fatty acids, some as part of the same compound (e.g., compounds **1**, **7**), from bacteria is however unusual. The HRMS^2^ data permitted to confirm and to highlight three types of (lower) fatty acyl moieties (i.e., *iso*-tetradecanoic acid (**1**), *iso*-pentadecanoic acid (**4**), *iso*-pentadecenoic acid (**2**, **3**, **5**, **6**, **7**) and in all seven compounds isolated. The chain length of the (upper) 3-hydroxy *iso*-fatty acid amide was generally C17, except for the compound **6** (C16). These information combined with detailed NMR data confirmed the compound identifications.

## 4. Materials and Methods 

### 4.1. General Experimental Procedures

Optical rotation measurements were conducted on a Jasco P-2000 polarimeter (Jasco, Pfungstadt, Germany). IR spectra were recorded on a PerkinElmer Spectrum Two FT-IR spectrometer (PerkinElmer, Boston, MA, USA). NMR spectra were obtained on a Bruker AV 600 spectrometer (600 and 150 MHz for ^1^H and ^13^C NMR, respectively, Bruker^®^, Billerica, MA, USA) equipped with a Z-gradient triple resonance cryo-probehead. The residual solvent signals of chloroform-*d* were used as internal references (*δ*_H_ 7.26 and *δ*_C_ 77.2). High-resolution mass spectrometry and fragmentation data were recorded using a Waters Xevo G2-XS QTof Mass Spectrometer (Waters^®^, Milford, MA, USA) coupled to a Waters Acquity UPLC system (Waters^®^, Milford, MA, USA). Crude extract fractionation was performed using Chromabond SPE C18 column cartridges (Macherey-Nagel, Duren, Germany). HPLC separations were performed on a VWR Hitachi Chromaster system (VWR International, Allison Park, PA, USA) consisting of a 5430 diode array detector (VWR International, Allison Park, PA, USA), a 5310 column oven, a 5260 autosampler and a 5110 pump combined in parallel with a VWR Evaporative Light Scattering Detector (ELSD 90, VWR International, Allison Park, PA, USA). The eluents used for HPLC separations were H_2_O (A) and MeCN (B). Routine HPLC separations were performed on semipreparative (Onyx, 10 mm × 100 mm, Phenomenex, Torrance, CA, USA) and analytic (Onyx, 3 mm × 100 mm, Phenomenex, Torrance, CA, USA) C18 Monolithic Phenomenex columns. MCC culture chips (Elga Europe, Milan, Italy and Hoekmine BV, Utrecht, The Netherlands) were fabricated as previously described using a photolithographic process to create 4500 microwells (180 µm diameter, 10 µm high walls) on a 36 mm × 8 mm (10 µm thick, pore size < 200 nm) wafer of porous aluminium oxide (General Electric, Frankfurt am Main, Germany) [5,6]. 

### 4.2. Isolation and Identification of Biological Material

The shallow water (50 cm depth) sediments were collected by using sterile 50 mL Falcon tubes in January 2014 during an expedition in the framework of National Program for Antarctic Research of Italy (PNRA) in the area of Edmonson Point, Antarctica. The isolation of the bacterium from Antartic sea sediment using the MCC will be described in detail in another communication. Briefly, the culture chips were sterilized by high intensity UV treatment for 30 min and were then placed on the flat surface of packed sediment in a Petri dish. Microcolonies growing after 1–3 weeks were recovered using a fine toothpick and were suspended in 50 µL of sterile water. Half of the resuspended colony was stored at −80 °C with 20% glycerol while the other half used to perform the identification via PCR of 16S rDNA genes. PCR products were then purified, sequenced and submitted to BLAST for the phylogenetic analysis. The 16S rRNA sequence of 1432 bp was compared to the NCBI nucleotide database by using BLASTn. The closest relative type strain is *Aequorivita antarctica* strain SW49T/ACAM640T (GenBank accession no: NR_025639.1), which shows a sequence similarity of 98% to our isolate and was isolated from under-ice seawater in Prydz Bay, Antarctica [13]. The 16S sequence was deposited in GenBank (accession number MH012204). A voucher specimen (23L) is housed at Institute of Protein Biochemistry, National Research Council, Italy.

### 4.3. Fermentation, Extraction and Isolation

Large-scale culture (10 L) was performed cultivating *Aequorivita* sp. in Marine Broth medium (Difco^TM^, Eysins, Switzerland) for 5 days at 20 °C at 200 rpm. The culture was centrifuged and pellet and supernatant were separately extracted with EtOAc. The EtOAc extract of the pellet was evaporated to dryness under vacuum, yielding 800 mg of dry residue. The crude EtOAc extract (380 mg) that showed moderate anti-MRSA activity was fractionated on a Chromabond SPE C18 cartridge. The elution with a 25% gradient of MeCN afforded 6 fractions. Anti-MRSA activity was tracked to fractions 5 (5 mg) and 6 (241 mg). Due to the low amounts of fraction 5, only the SPE fraction 6 was worked-up. This fraction was subjected to semi-prep. RP-HPLC chromatography (gradient of H_2_O:MeCN from 50:50 to 0:100 in 27 min, flow 4.0 mL/min) to yield compounds **6** (1.7 mg, *t*_R_ 21.8 min) and **7** (1.7 mg, *t*_R_ 22.6 min), along with other five fractions (A–E). The fraction A (7 mg) was then chromatographed a second time by RP-HPLC (isocratic mixture H_2_O:MeCN 1:9 in 10 min, flow 1.0 mL/min) to yield **3** (1.0 mg, *t*_R_ 7.0 min) in a pure state. The fraction B (6 mg) was further purified by RP-HPLC (isocratic mixture H_2_O:MeCN 1:9 in 10 min, flow 1.0 mL/min) to give **5** (1.0 mg, *t*_R_ 7.3 min) and **1** (1.5 mg, *t*_R_ 8.2 min). The purification of the fraction C (5.6 mg) under the same RP-HPLC conditions afforded **2** (4.0 mg, *t*_R_ 10 min). Finally, the fraction D (3 mg) was rechromatographed by RP-HPLC (gradient of H_2_O:MeCN from 50:50 to 0:100 in 8 min, flow 1.0 mL/min) affording **4** (1.0 mg, *t*_R_ 10 min). 

### 4.4. LC-HRMS^2^

Experiments were performed using a Waters Xevo G2-XS QTof Mass Spectrometer coupled to a Waters Acquity UPLC I-Class system. The LC-MS analysis was performed using an Acquity UPLC HSS T3 column (High Strength Silica C18, 1.8 µm, 2.1 mm × 100 mm, Waters^®^, Milford, MA, USA), maintained at 40 °C, operating a linear gradient H_2_O:MeCN (both containing 0.1% of formic acid) from 1% MeCN to 100% MeCN over 15 min, keeping 100% MeCN for 3 min and reconditioning of the column during 3 min (flow rate 0.6 mL/min). All the high-resolution mass spectra were recorded in the positive-ion mode in a mass range from *m*/*z* 50 to 1600. MS parameters were: spray voltage of 0.8 kV, source temperature 150 °C, desolvation temperature 650 °C, cone gas flow 50 L/Hr and desolvation gas flow 1200 L/Hr. Data were collected in the data-dependent acquisition (DDA) mode and targeted MS/MS acquisition mode was performed for each pure compounds isolated. A collision energy ramp from 30 to 80 eV was applied. Acquisition and data analyses were carried out with the MassLynx software (version 4.1, Waters^®^, Milford, MA, USA). Multiple commercial and public databases (MarinLit, Dictionary of Natural Products, PubChem, Chemspider, Raleigh, NC, USA) were used for dereplication and annotation of known compounds.

*R-(+)-N-[15-methyl-3-(12-methyltridecanoyloxy)-hexadecanoyl]glycine (***1***)*: yellow oil; ${\left[\alpha \right]}_{D}^{\mathrm{22}}$ +45 (*c* 1.3, CHCl_3_); IR (film) *v*_max_ 3329, 2923, 2853, 1731, 1649, 1465, 1179 cm^−1^; ^1^H NMR (CDCl_3_, 600 MHz) and ^13^C NMR (CDCl_3_, 150 MHz) Table 1 and Table 2; HRESIMS found *m*/*z* [M + Na]^+^ 576.4603; C_33_H_63_NO_5_Na, requires 576.4604.

*R-(+)-N-[15-methyl-3-(13-methyl-4Z-tetradecenoyloxy)-hexadecanoyl]glycine methyl ester (***2***)*: yellow oil; ${\left[\alpha \right]}_{D}^{\mathrm{22}}$ +8.5 (*c* 1.3, CHCl_3_); IR (film) *v*_max_ 3386, 2924, 2853, 1733, 1645, 1466, 1202 cm^−1^; ^1^H NMR (CDCl_3_, 600 MHz) and ^13^C NMR (CDCl_3_, 150 MHz) Table 1 and Table 2; HRESIMS found *m*/*z* [M + Na]^+^ 602.4767; C_35_H_65_NO_5_Na requires 602.4760.

*N-[N-[3R-15-methyl-3-(13-methyl-4Z-tetradecenoyloxy)-hexadecanoyl]glycyl]-l-serine methyl ester (***3***)*: yellow oil; ${\left[\alpha \right]}_{D}^{\mathrm{22}}$ +45 (*c* 1.3, CHCl_3_); IR (film) *v*_max_ 3360, 2923, 2853, 1734, 1657, 1544, 1465, 1366, 1205, 1177, 1035 cm^−1^; ^1^H NMR (CDCl_3_, 600 MHz) and ^13^C NMR (CDCl_3_, 150 MHz) Table 1 and Table 2; HRESIMS found *m*/*z* [M + Na]^+^ 689.5085; C_38_H_70_N_2_O_7_Na requires 689.5081.

### 4.5. Anti-MRSA Assay

Antibacterial assay was performed using the clinically relevant bacterial test strain *Staphylococcus aureus* (methillicin-resistant, DSM 18827). It was cultivated in Trypticase soy broth (1.2% Trypticase soy broth, 0.5% NaCl) overnight and diluted to an OD600 of 0.01. 40 mg mL^−1^ DMSO stock solution of the compound was diluted with medium to gain the desired test concentrations. In addition, 10.5 µL of the compound solution and 200 µL of the test strain cell suspension were transferred in a 96-well microtiter plate and incubated for 5 h at 37 °C and 200 rpm. After the addition of 10 µL of a resazurin solution as the detective reagent (0.3 mg mL^−1^ in phosphate-buffered saline), the incubation was continued for another 5 min. To evaluate cell viability, the reduction of resazurin to resorufin was determined by measuring the intensity of fluorescence at 560Ex/590Em in a Tecan Infinite M200 plate reader (Männedorf, Switzerland). The IC_50_ values were calculated by Excel (version 2013, Microsoft, Redmond, Washington, DC, USA) as the concentration that showed 50% inhibition of viability on the basis of a negative control (no compound). Chloramphenicol was used as a positive control.

## 5. Conclusions

In summary, LC-MS^2^-based dereplication in combination with a traditional MRSA-activity guided fractionation allowed the accelerated work-up of the Antarctic Gram-negative bacterium *Aequorivita* sp. that was rapidly isolated by an innovative culture chip technique. We report herein the isolation, structural elucidation and moderate antimicrobial activity of natural linear aminolipids. This is the first chemical and bioactivity study performed on an *Aequorivita* species.

## Figures and Tables

**Table 1:** ^1^H NMR data of compounds **1**–**3** (600 MHz, CDCl_3_).

Position	1	2	3
	*δ*_H_, Mult. (*J* in Hz)	*δ*_H_, Mult. (*J* in Hz)	*δ*_H_, Mult. (*J* in Hz)
2	2.54, m	2.51, m	2.50, m
3	5.16, m	5.17, m	5.17, m
4	1.62 ^a^	1.60, m	1.60, m
5	1.30 ^a^	1.30 ^a^	1.30 ^a^
6–13	1.25 ^a^	1.25 ^a^	1.25 ^a^
14	1.14, m	1.14, m	1.14, m
15	1.51, m	1.51, m	1.51, m
16	0.86, d (6.7)	0.86, d (6.7)	0.86, d (6.7)
17	0.86, d (6.7)	0.86, d (6.7)	0.86, d (6.7)
2′	2.30, t (7.5)	2.34 ^a^	2.37 ^a^
3′	1.60 ^a^	2.34 ^a^	2.36 ^a^
4′	1.28 ^a^	5.30, m	5.30, m
5′	1.25 ^a^	5.40, m	5.42, m
6′	1.25 ^a^	2.02, m	2.04, m
7′	1.25 ^a^	1.33, m	1.33, m
8′–10′	1.25 ^a^	1.25, m	1.25, m
11′	1.14, m	1.25 ^a^	1.25 ^a^
12′	1.51, m	1.14, m	1.14, m
13′	0.86, d (6.7)	1.51, m	1.51, m
14′	0.86, d (6.7)	0.86, d (6.7)	0.86, d (6.7)
15′		0.86, d (6.7)	0.86, d (6.7)
2′′	4.07, d (5.0)	4.03, d (5.3)	4.03, d (5.3)
2′′′			4.66, m
3′′′			4.00 ^a^ 3.96 ^a^
OCH_3_		3.75, s	3.75, s
(Gly)NH	6.38, brt (5.3)	6.25, brt (5.0)	6.38, t (4.5)
(Ser)NH			6.90, d (7.7)

^a^ Overlapped with other signals.

**Table 2:** ^13^C NMR data of compounds **1**–**3** (150 MHz, CDCl_3_).

Position	1 ^a^	2	3
	*δ*_C_, Type	*δ*_C_, Type	*δ*_C_, Type
1	170.5, C	169.9, C	170.8, C
2	41.4, CH_2_	41.2, CH_2_	41.7, CH_2_
3	71.1, CH	71.3, CH	71.5, CH
4	34.1, CH_2_	34.3, CH_2_	34.2, CH_2_
5	25.4, CH_2_	29.5, CH_2_	29.6, CH_2_
6–13	29.0, CH_2_	29.0, CH_2_	29.0, CH_2_
14	39.1, CH_2_	39.1, CH_2_	39.1, CH_2_
15	27.8, CH	27.8, CH	27.8, CH
16	22.7, CH_3_	22.7, CH_3_	22.7, CH_3_
17	22.7, CH_3_	22.7, CH_3_	22.7, CH_3_
1′	173.7, C	173.9, C	173.7, C
2′	34.5, CH_2_	34.4, CH_2_	34.4, CH_2_
3′	24.9, CH_2_	22.7, CH_2_	22.6, CH_2_
4′	29.0, CH_2_	127.2, CH	127.1, CH
5′	29.0, CH_2_	131.6, CH	131.7, CH
6′	29.0, CH_2_	27.1, CH_2_	27.1, CH_2_
7′	29.0, CH_2_	29.6, CH_2_	29.5, CH_2_
8′–10′	29.0, CH_2_	29.0, CH_2_	29.0, CH_2_
11′	39.1, CH_2_	29.0, CH_2_	29.0, CH_2_
12′	27.8, CH	39.1, CH_2_	39.1, CH_2_
13′	22.7, CH_3_	27.8, CH	27.8, CH
14′	22.7, CH_3_	22.7, CH_3_	22.7, CH_3_
15′		22.7, CH_3_	22.7, CH_3_
1′′	171.1, C	170.2, C	170.0, C
2′′	41.1, CH_2_	41.2, CH_2_	41.1, CH_2_
1′′′			170.6, C
2′′′			54.8, CH_2_
3′′′			62.7, CH
OCH_3_		52.0, CH_3_	52.0, CH_3_

^a^ Extracted from HSQC and HMBC spectra.

**Table 3:** In vitro anti-MRSA activity of the EtOAc pellet extract and compounds **1**–**7**. Reference drug: chloramphenicol.

Sample	IC_50_ Value (µg/mL)
EtOAc extract	120
**1**	58
**2**	145
**3**	>200
**4**	22
**5**	93
**6**	>200
**7**	>200
Reference drug	2.89

## Supplementary Materials

The following are available online at http://www.mdpi.com/1660-3397/16/6/187/s1, NMR, HRESIMS and MS/MS spectra of compounds **1**–**3**, and HRESI-MS/MS spectra of the known compounds **4**–**7**.
