# Supplementary material for: Linear Aminolipids with Moderate Antimicrobial Activity from the Antarctic Gram-Negative Bacterium Aequorivita sp
Source: Mar Drugs. 2018 May 28;16(6):187. doi: 10.3390/md16060187 (PMC6025266; doi:10.3390/md16060187)
Supplement: Supplementary file 1 [file marinedrugs-16-00187-s001.pdf]

SUPPLEMENTARY MATERIALS

Linear Aminolipids with Moderate Antimicrobial Activity from the Antarctic Gram-Negative Bacterium *Aequorivita* sp.

Giuseppina Chianese[1], Fortunato Palma Esposito[2], Delphine Parrot[1], Colin Ingham[3], Donatella de Pascale[2] and Deniz Tasdemir[1,4,*]

1 GEOMAR Centre for Marine Biotechnology (GEOMAR-Biotech), Research Unit Marine Natural Products Chemistry, GEOMAR Helmholtz Centre for Ocean Research Kiel, Am Kiel-Kanal 44, Kiel 24106, Germany; g.chianese@unina.it; dparrot@geomar.de; dtasdemir@geomar.de

2 Institute of Protein Biochemistry, National Research Council, Via P. Castellino, 111, I-80131 Naples, Italy; f.palma@ibp.cnr.it; d.depascale@ibp.cnr.it

3 Hoekmine BV, Utrecht 3584 CS, The Netherlands; colinutrecht@gmail.com

4 Kiel University, Christian-Albrechts-Platz 4, 24118, Kiel, Germany

* Correspondence: dtasdemir@geomar.de; Tel.: +49-431-600-4430

Figure No	Page No
**Figure S1**. ^{1}H NMR (600 MHz, $CDCl_3$) spectrum of compound **1**	**3**
**Figure S2.** COSY NMR (600 MHz, $CDCl_3$) spectrum of compound **1**	**3**
**Figure S3.** HMBC NMR (600 MHz, $CDCl_3$) spectrum of compound **1**	**4**
**Figure S4.** HSQC NMR (600 MHz, $CDCl_3$) spectrum of compound **1**	**4**
**Figure S5.** HRESIMS/MS spectrum in positive mode of compound **1**	**5**
**Figure S6**. ^{1}H NMR (600 MHz, $CDCl_3$) spectrum of compound **2**	**6**
**Figure S7.** ^{13}C NMR (150 MHz, $CDCl_3$) spectrum of compound **2**	**6**
**Figure S8.** COSY NMR (600 MHz, $CDCl_3$) spectrum of compound **2**	**7**
**Figure S9**. HMBC NMR (600 MHz, $CDCl_3$) spectrum of compound **2**	**7**
**Figure S10.** HSQC NMR (600 MHz, $CDCl_3$) spectrum of compound **2**	**8**
**Figure S11.** NOESY NMR (600 MHz, $CDCl_3$) spectrum of compound **2**	**8**
**Figure S12**. HRESIMS and MS/MS spectra in positive mode of compound **2**	**9**
**Figure S13**. ^{1}H NMR (600 MHz, $CDCl_3$) spectrum of compound **3**	**10**
**Figure S14**. ^{13}C NMR (150 MHz, $CDCl_3$) spectrum of compound **3**	**10**
**Figure S15.** COSY NMR (600 MHz, $CDCl_3$) spectrum of compound **3**	**11**
**Figure S16.** HMBC NMR (600 MHz, $CDCl_3$) spectrum of compound **3**	**11**
**Figure S17.** HSQC NMR (600 MHz, $CDCl_3$) spectrum of compound **3**	**12**
**Figure S18.** NOESY NMR (600 MHz, $CDCl_3$) spectrum of compound **3**	**12**
**Figure S19.** HRESIMS and MS/MS spectra in positive mode of compound **3**	**13**
**Figure S20.** Annotated HRESI-MS/MS spectra in positive mode of the known compounds **4-7**	**14-15**

Figure S1. ^{1}H NMR (600 MHz, $CDCl_3$) spectrum of compound **1**

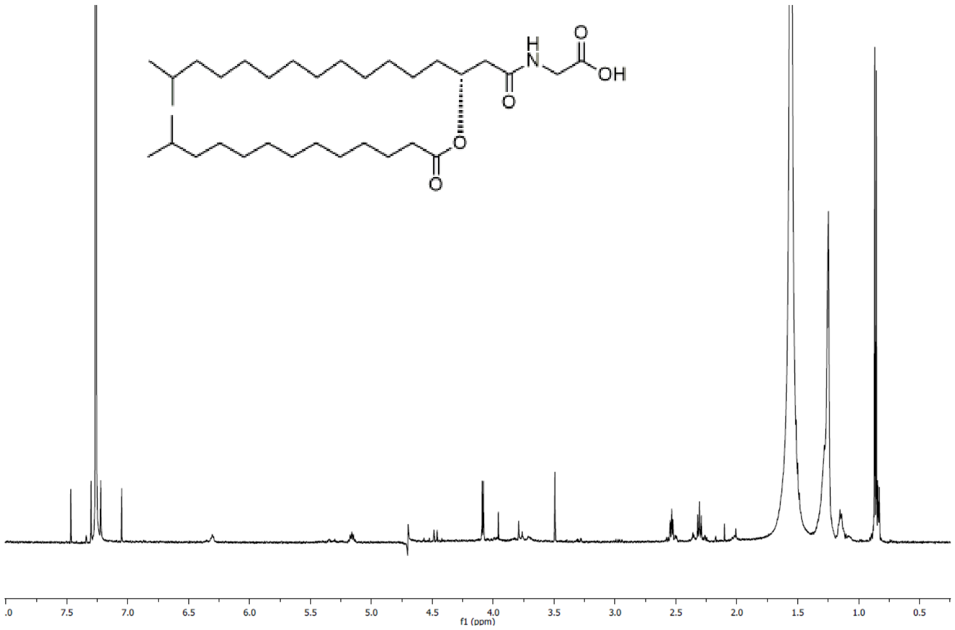

Figure S2. COSY NMR (600 MHz, $CDCl_3$) spectrum of compound **1**

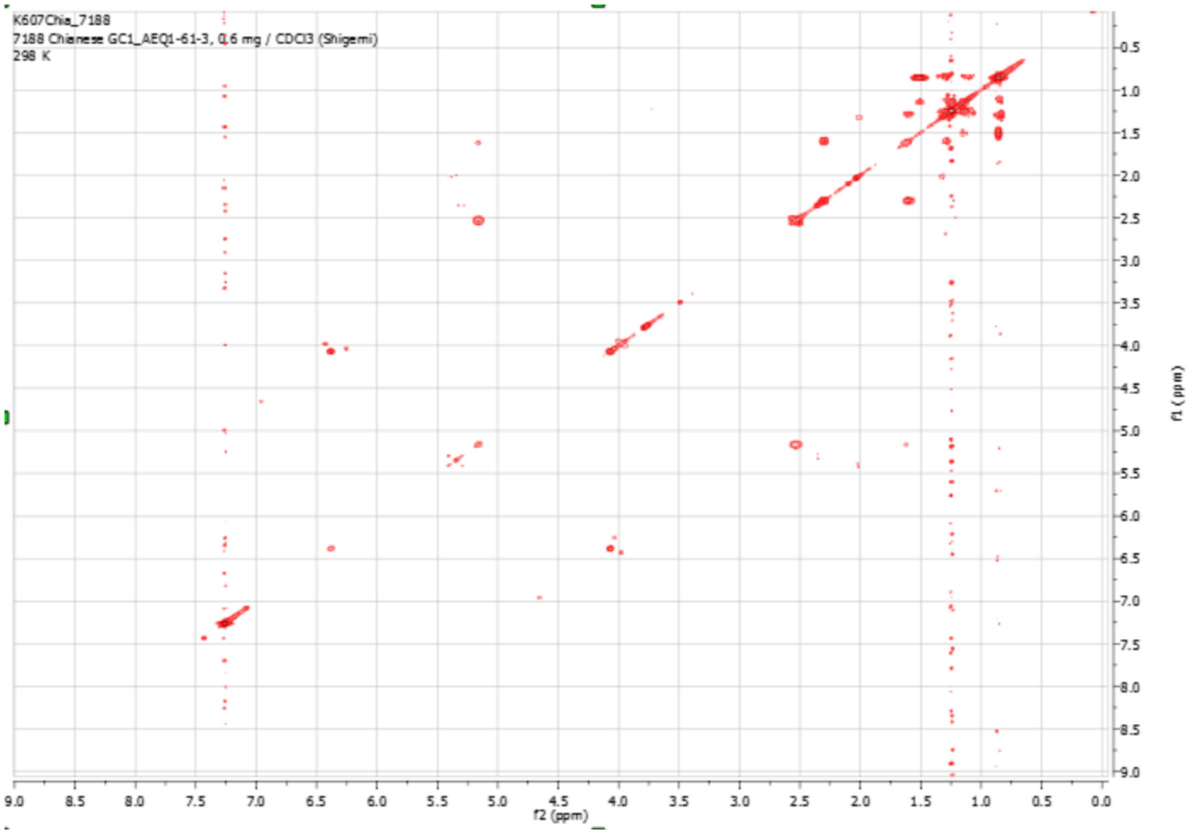

Figure S3. HMBC NMR (600 MHz, $CDCl_3$) spectrum of compound **1**

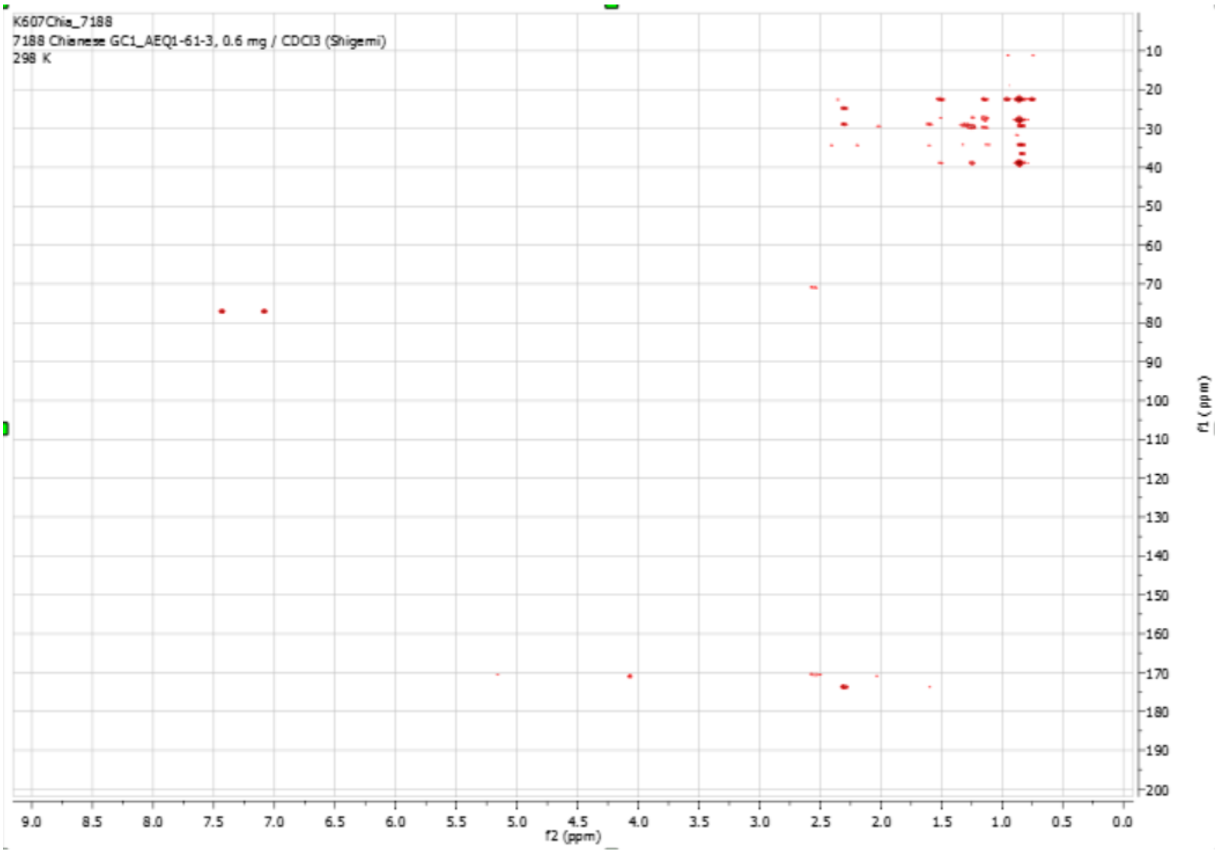

Figure S4. HSQC NMR (600 MHz, $CDCl_3$) spectrum of compound **1**

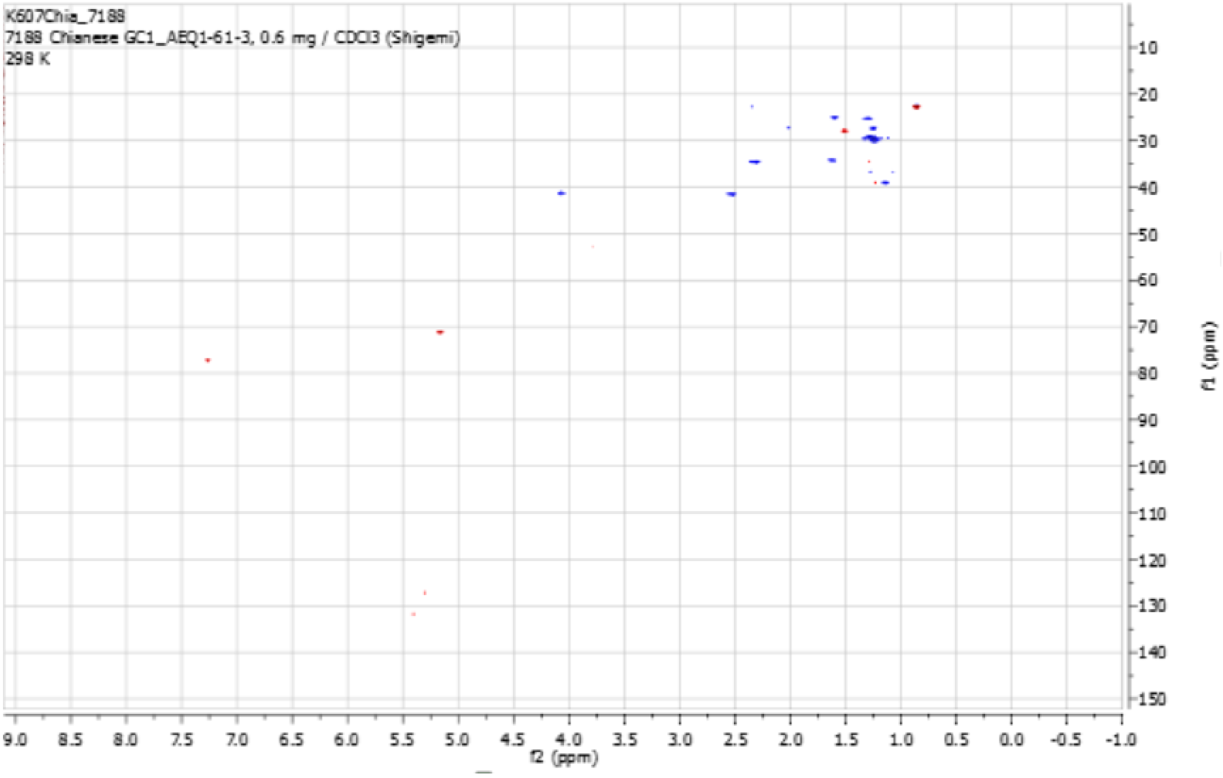

Figure S5. HRESIMS and MS/MS spectra in positive mode of compound **1**

(+)-HRESIMS

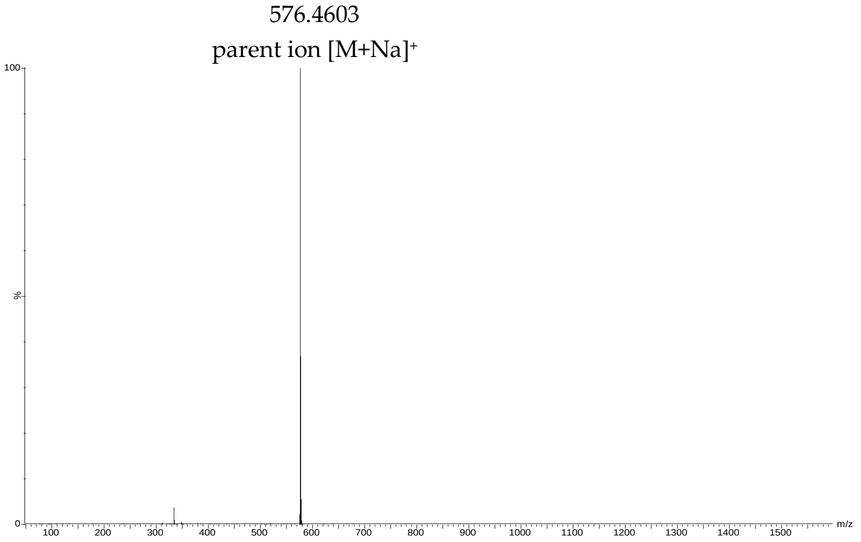

(+)-HRESIMS/MS

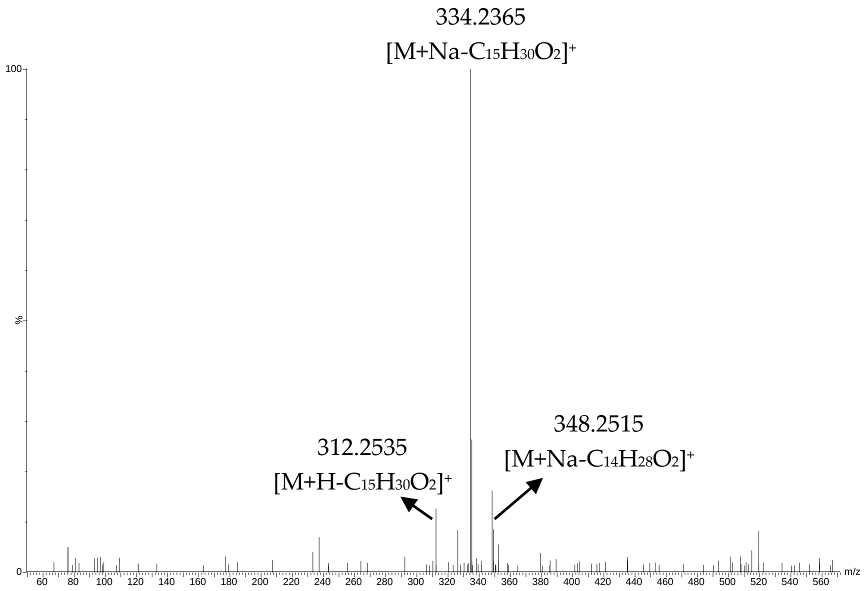

Figure S6. ^{1}H NMR (600 MHz, $CDCl_3$) spectrum of compound **2**

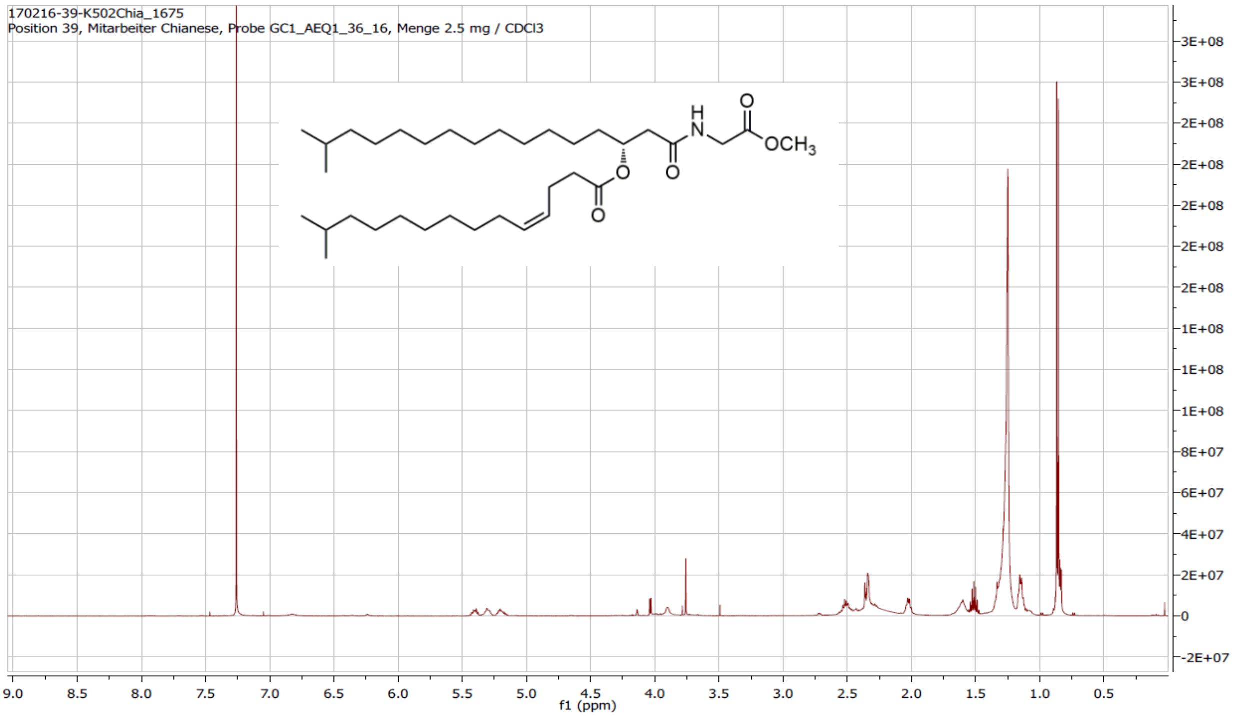

Figure S7. ^{13}C NMR (150 MHz, $CDCl_3$) spectrum of compound **2**

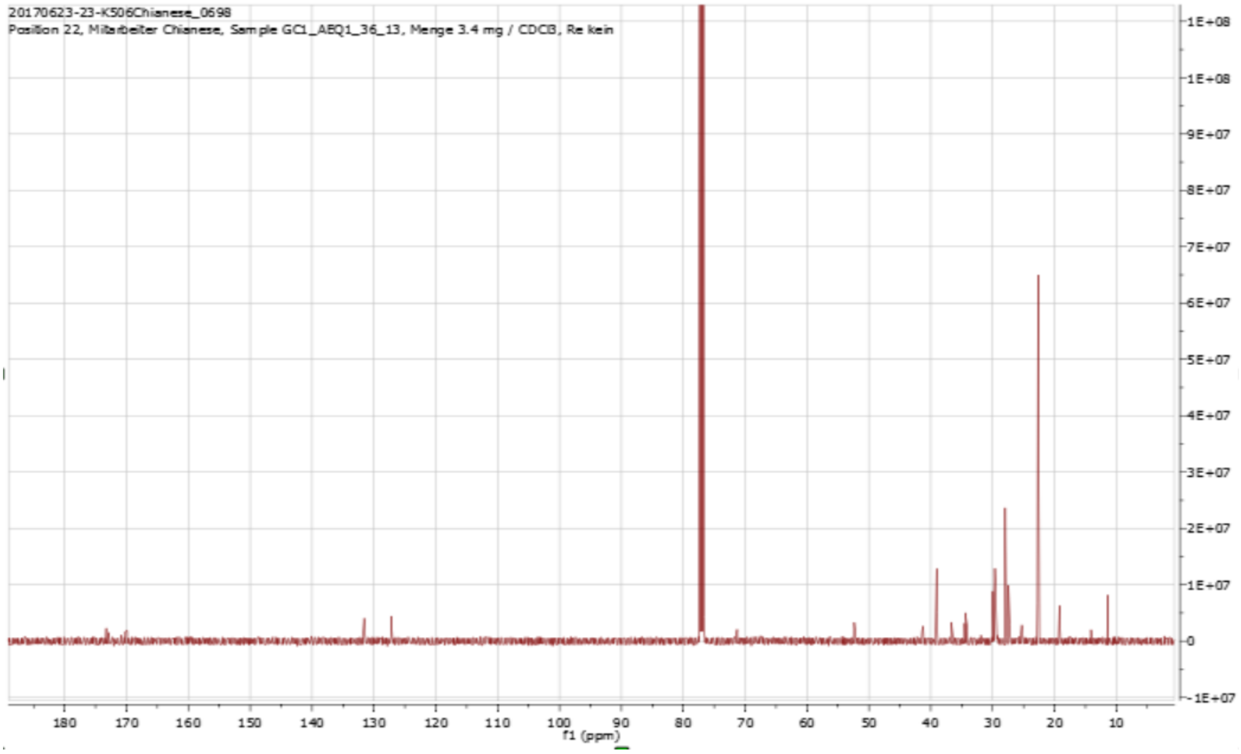

Figure S8. COSY NMR (600 MHz, $CDCl_3$) spectrum of compound **2**

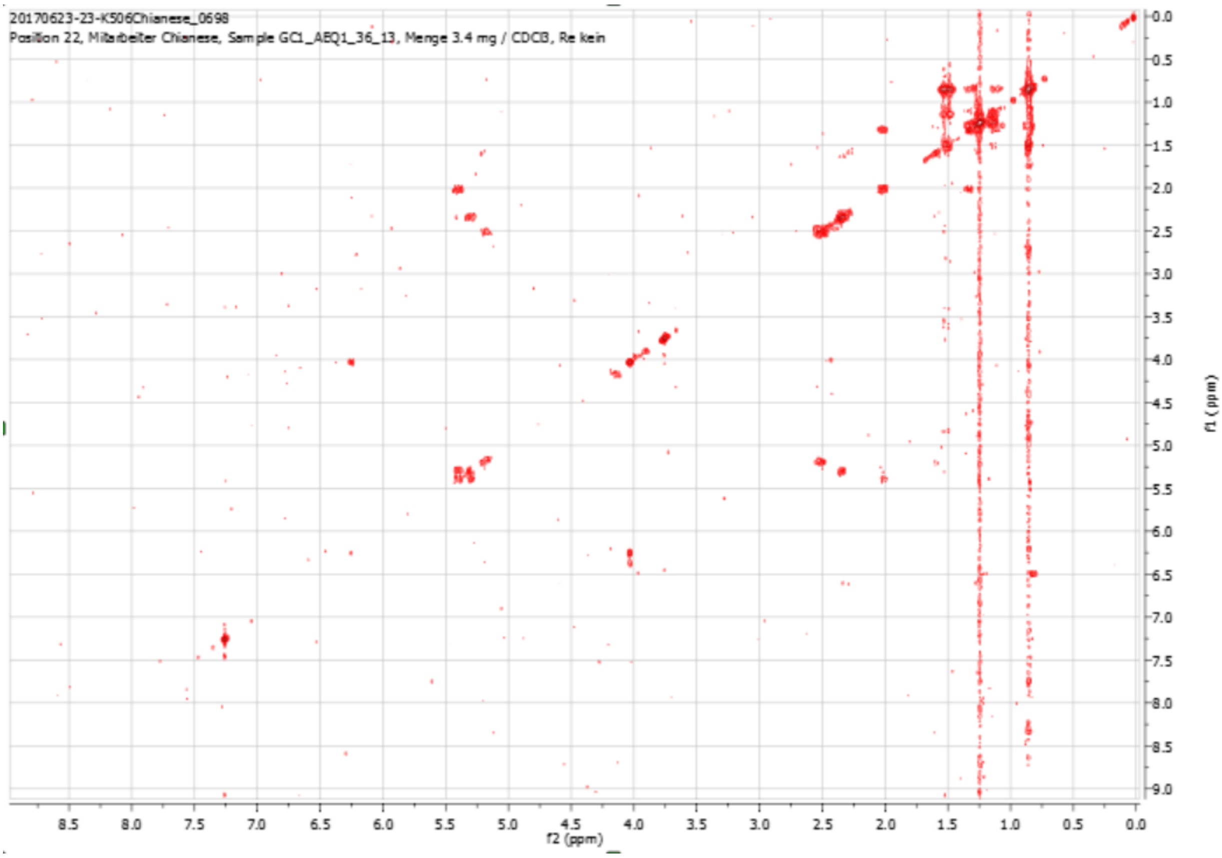

Figure S9. HMBC NMR (600 MHz, $CDCl_3$) spectrum of compound **2**

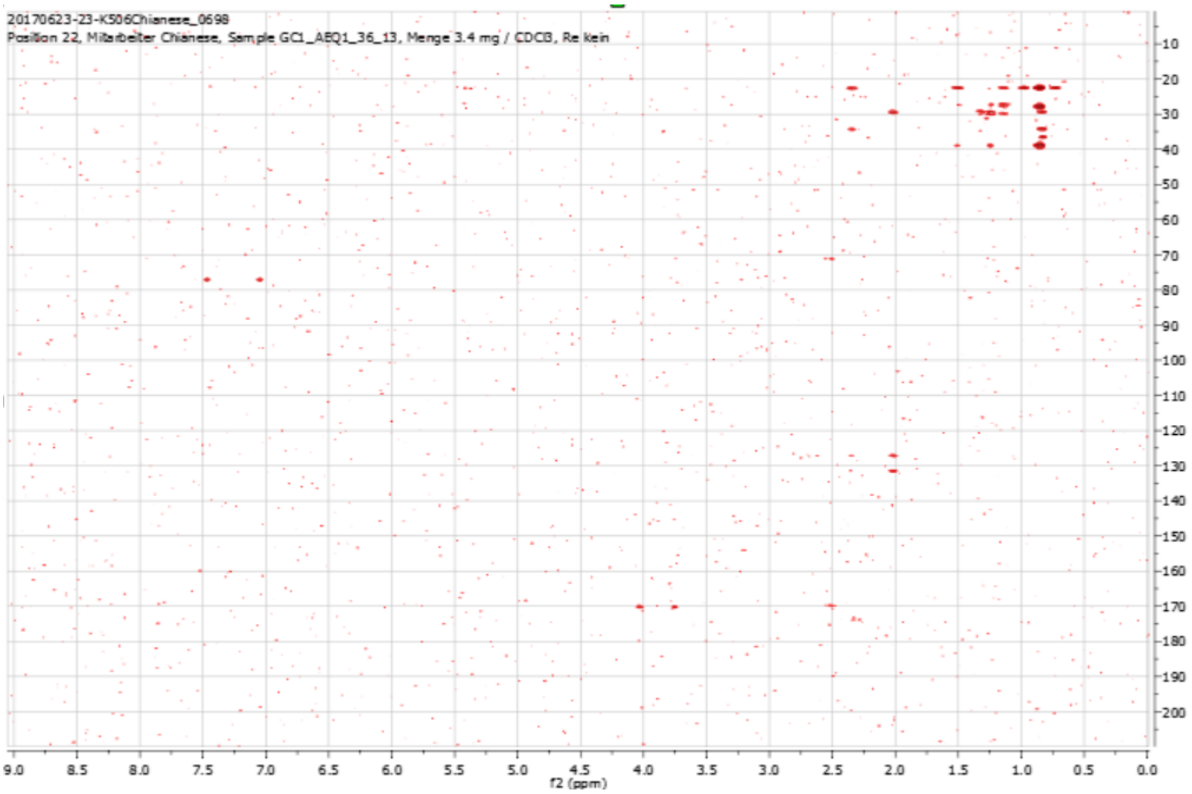

Figure S10. HSQC NMR (600 MHz, $CDCl_3$) spectrum of compound **2**

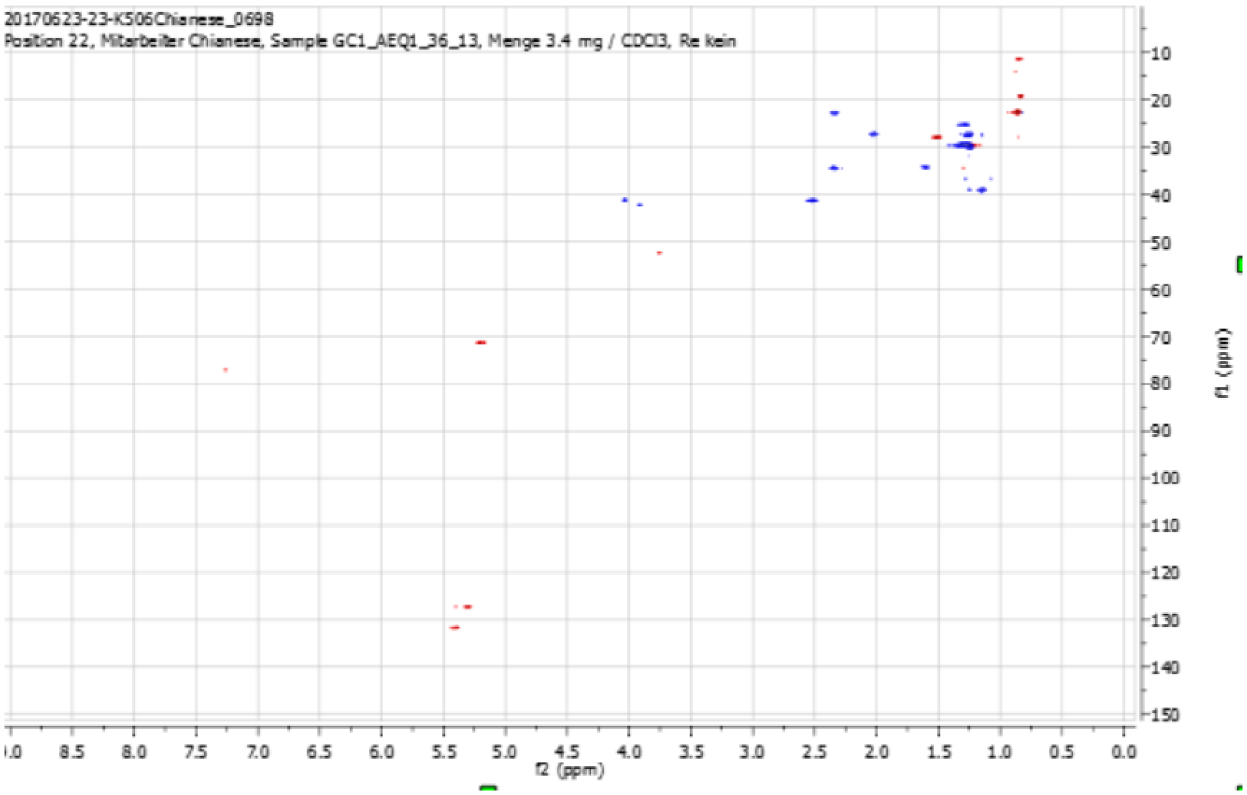

Figure S11. NOESY NMR (600 MHz, $CDCl_3$) spectrum of compound **2**

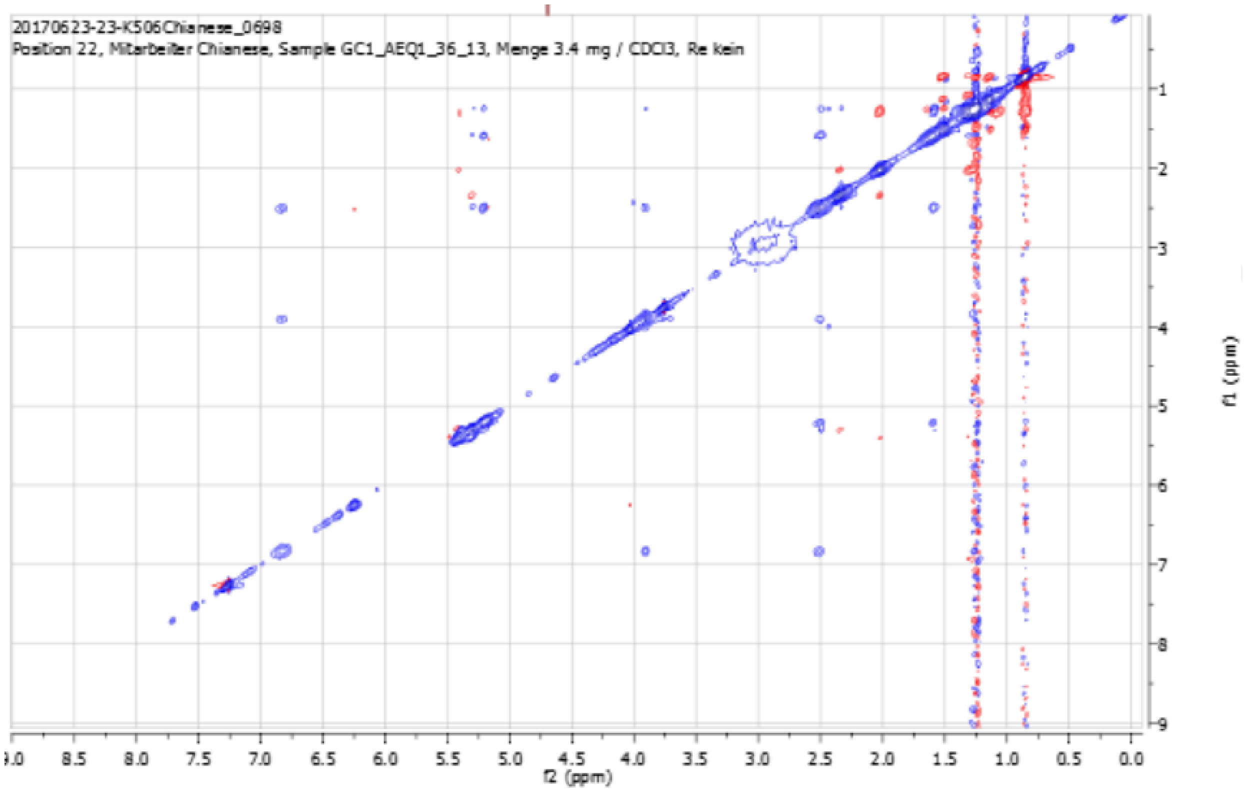

Figure S12. HRESIMS and MS/MS spectra in positive mode of compound **2**

(+)-HRESIMS

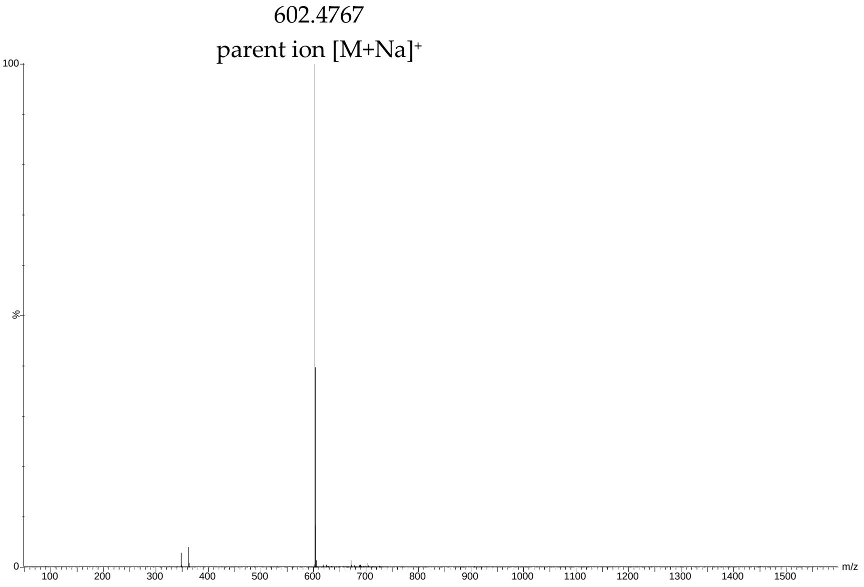

(+)-HRESIMS/MS

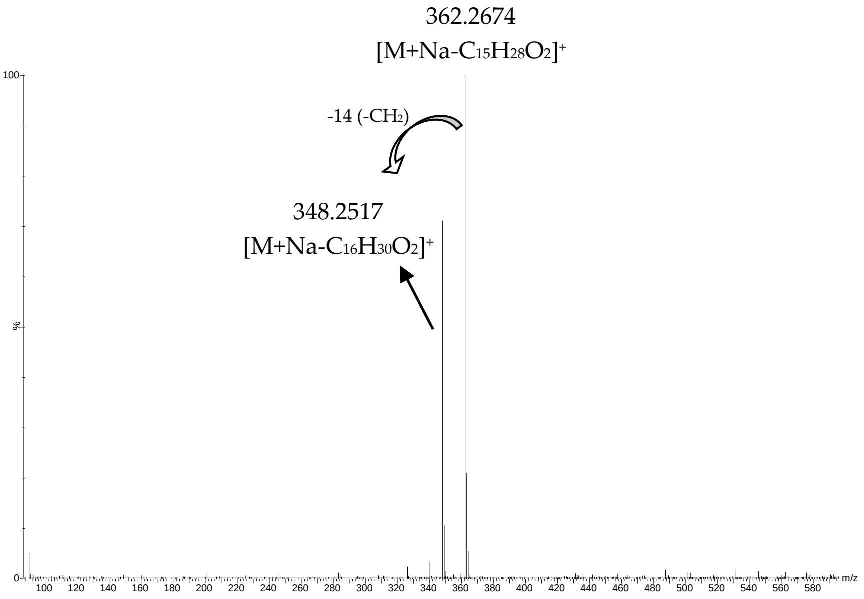

Figure S13. ^{1}H NMR (600 MHz, $CDCl_3$) spectrum of compound **3**

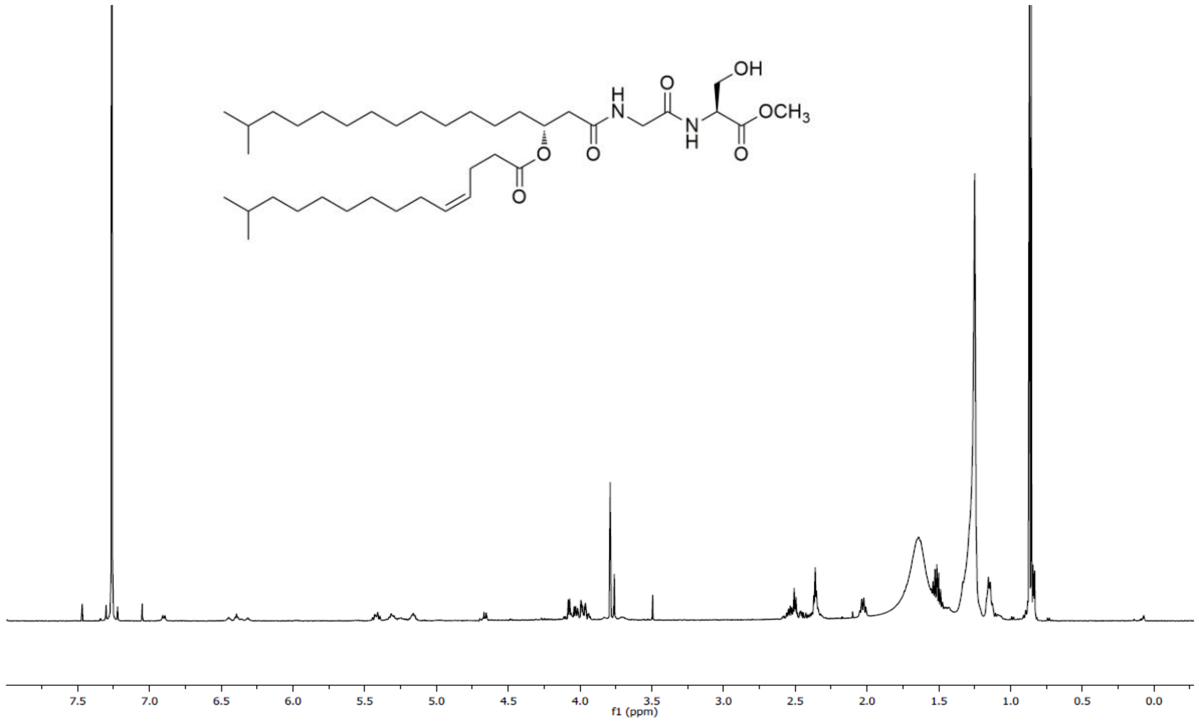

Figure S14. ^{13}C NMR (150 MHz, $CDCl_3$) spectrum of compound **3**

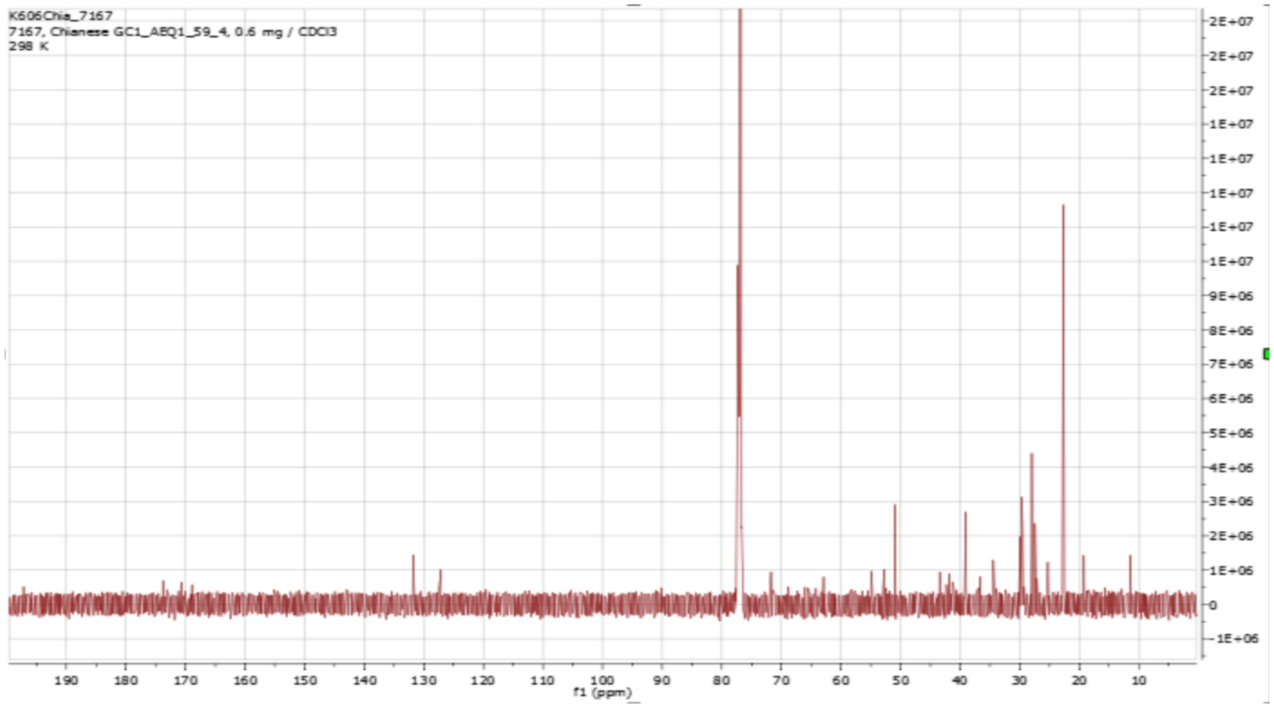

Figure S15. COSY NMR (600 MHz, $CDCl_3$) spectrum of compound **3**

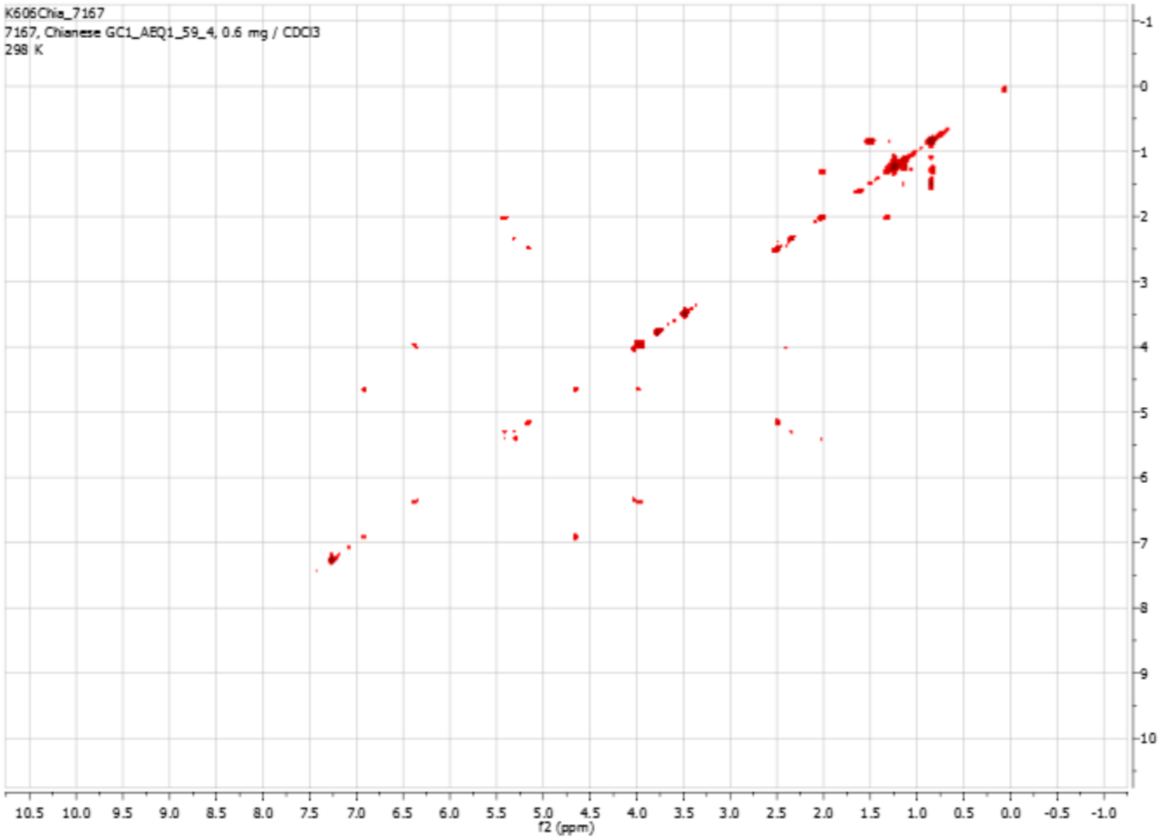

Figure S16. HMBC NMR (600 MHz, $CDCl_3$) spectrum of compound **3**

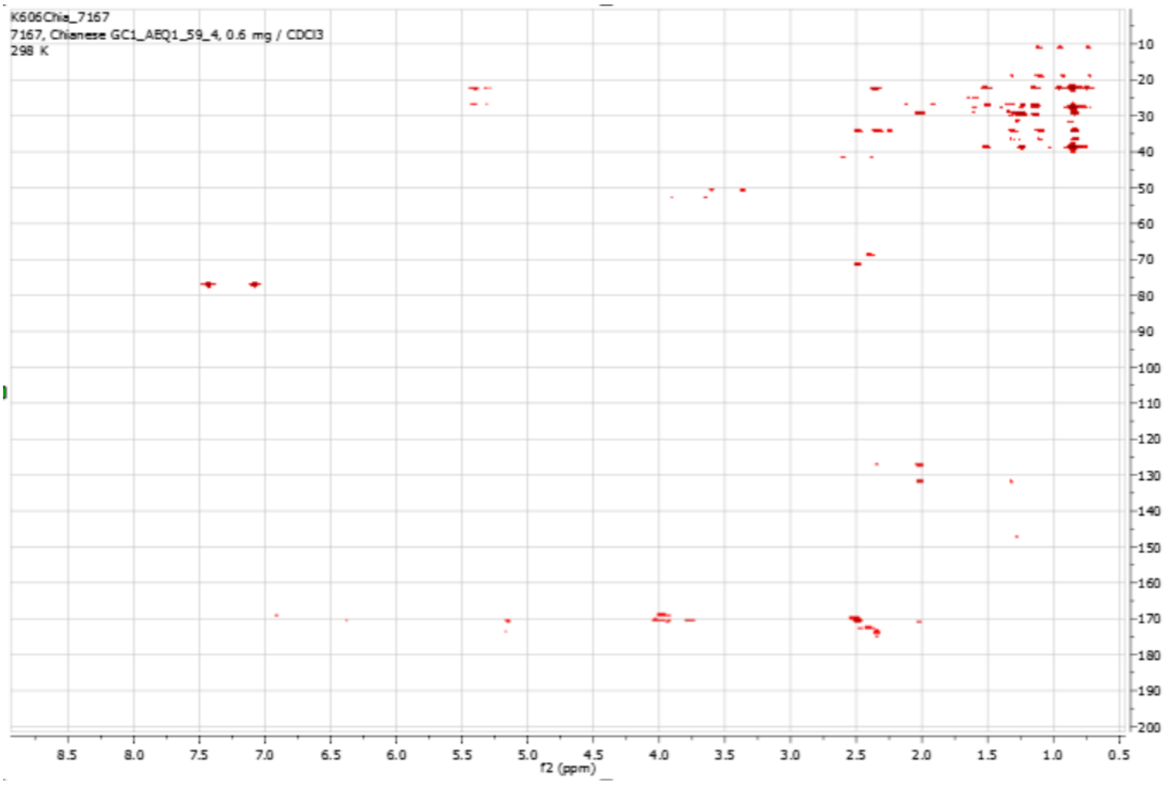

Figure S17. HSQC NMR (600 MHz, $CDCl_3$) spectrum of compound **3**

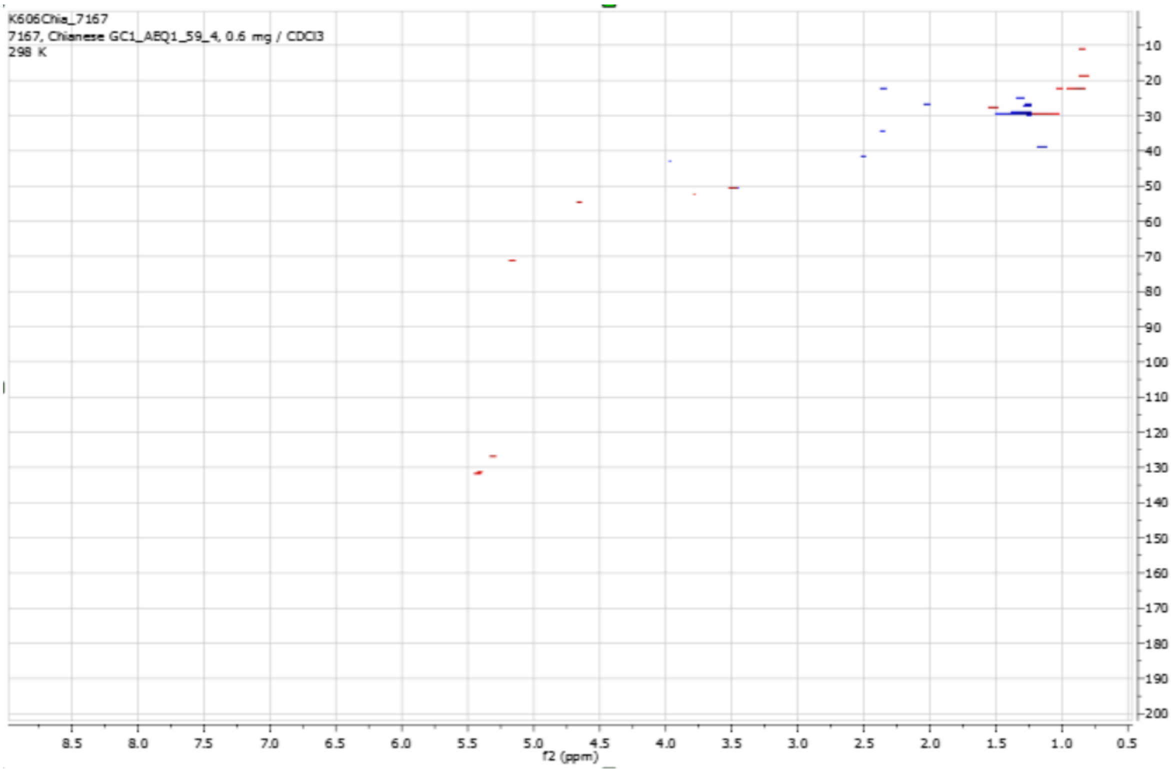

Figure S18. NOESY NMR (600 MHz, $CDCl_3$) spectrum of compound **3**

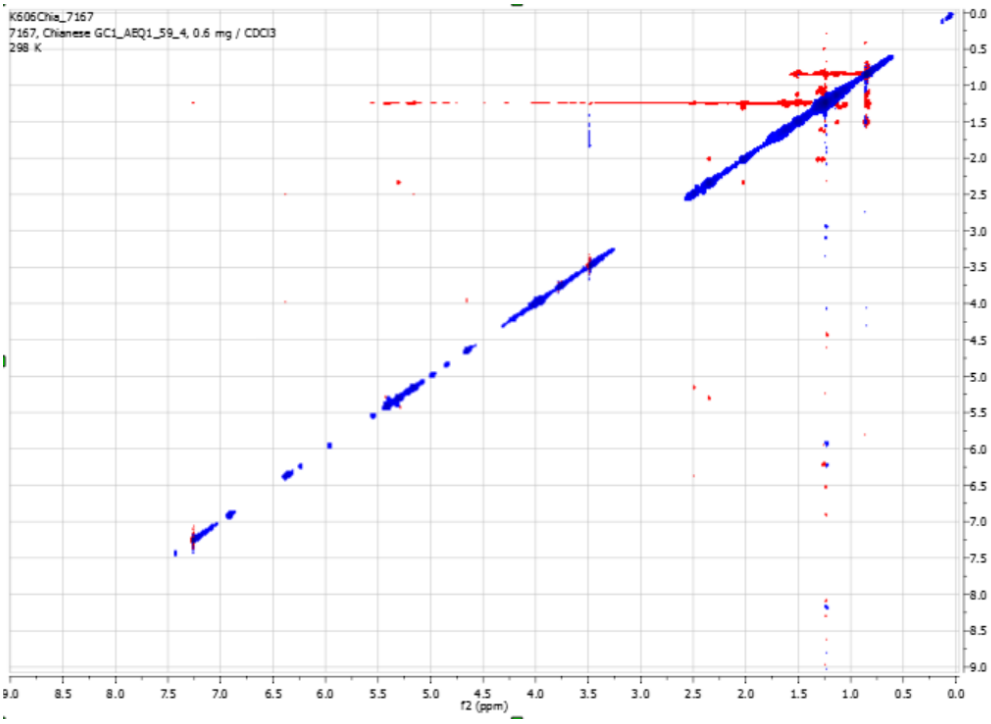

Figure S19. HRESIMS and MS/MS spectra in positive mode of compound **3**

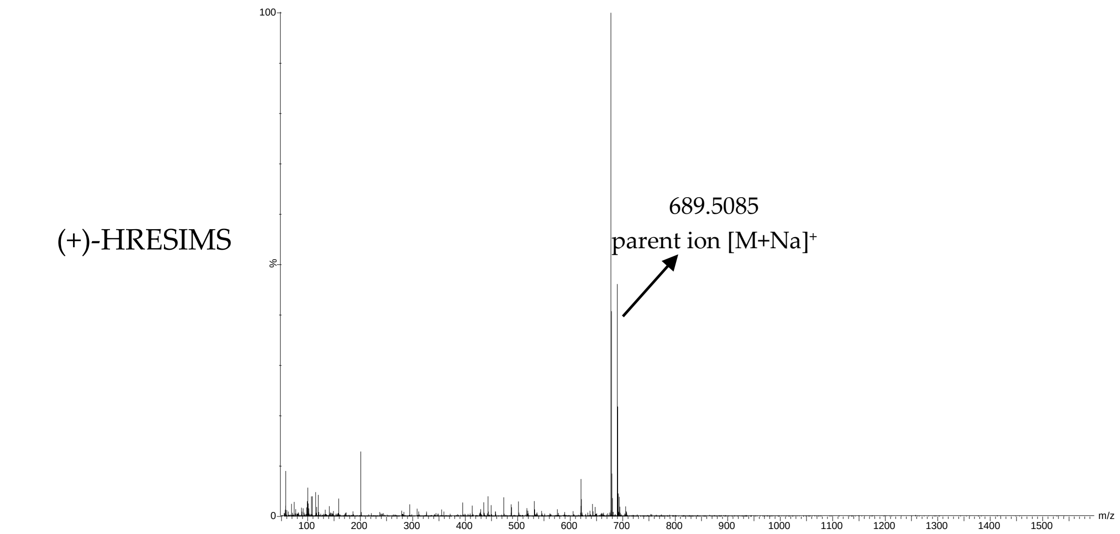

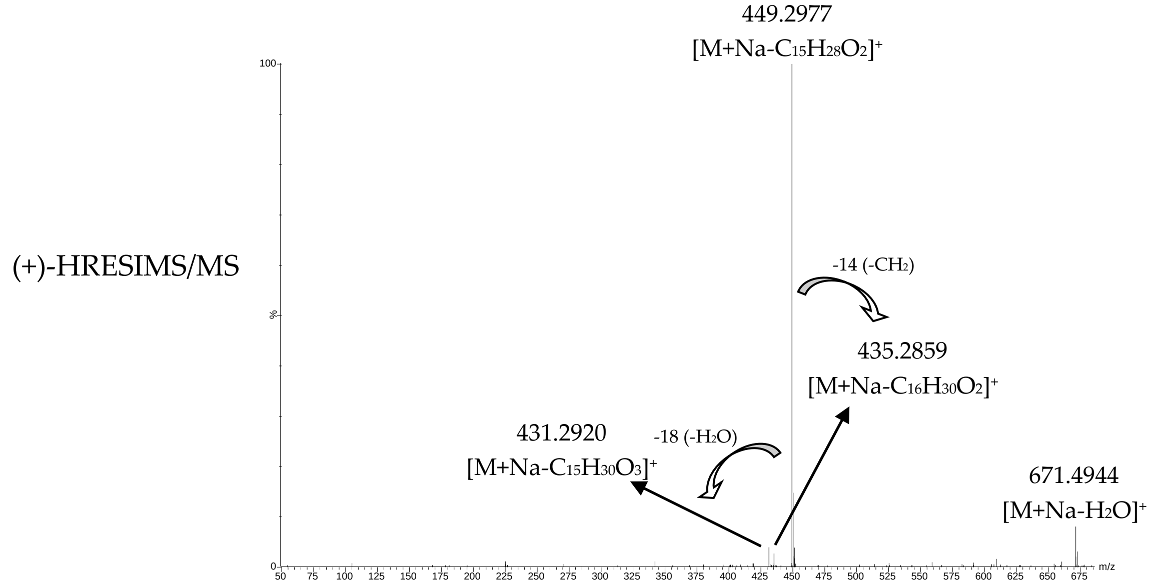

Figure S20. Annotated HRESI-MS/MS spectra in positive mode of the known compounds 4-7

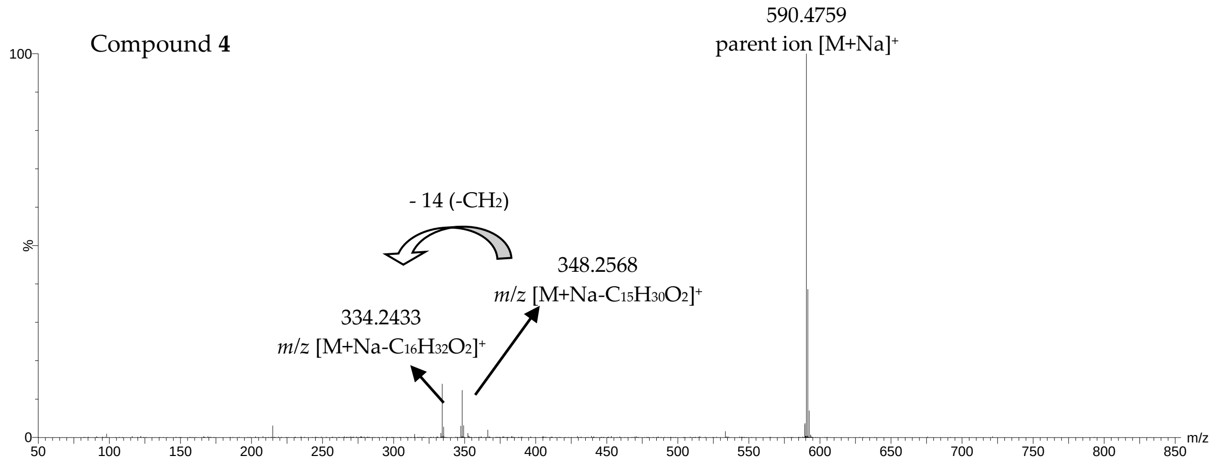

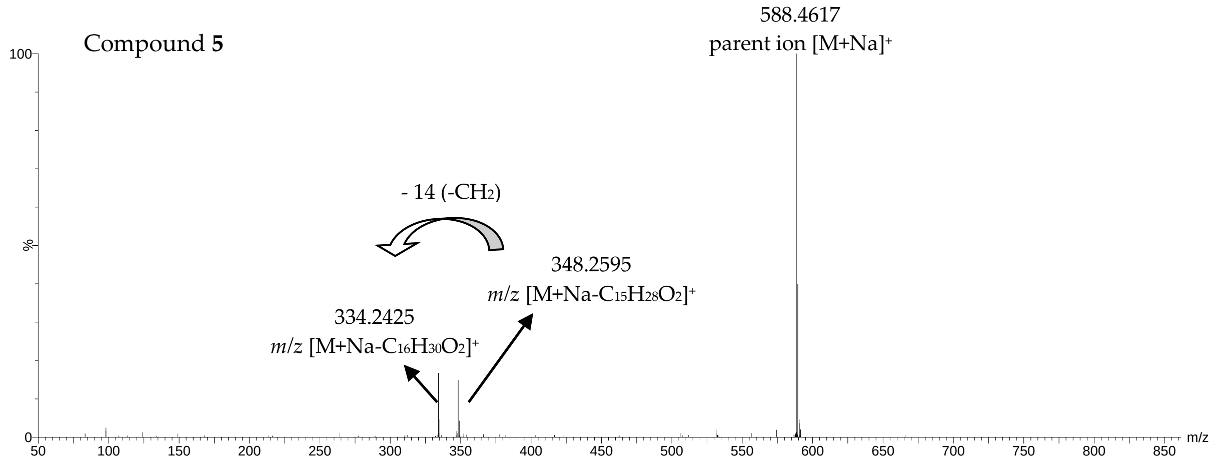

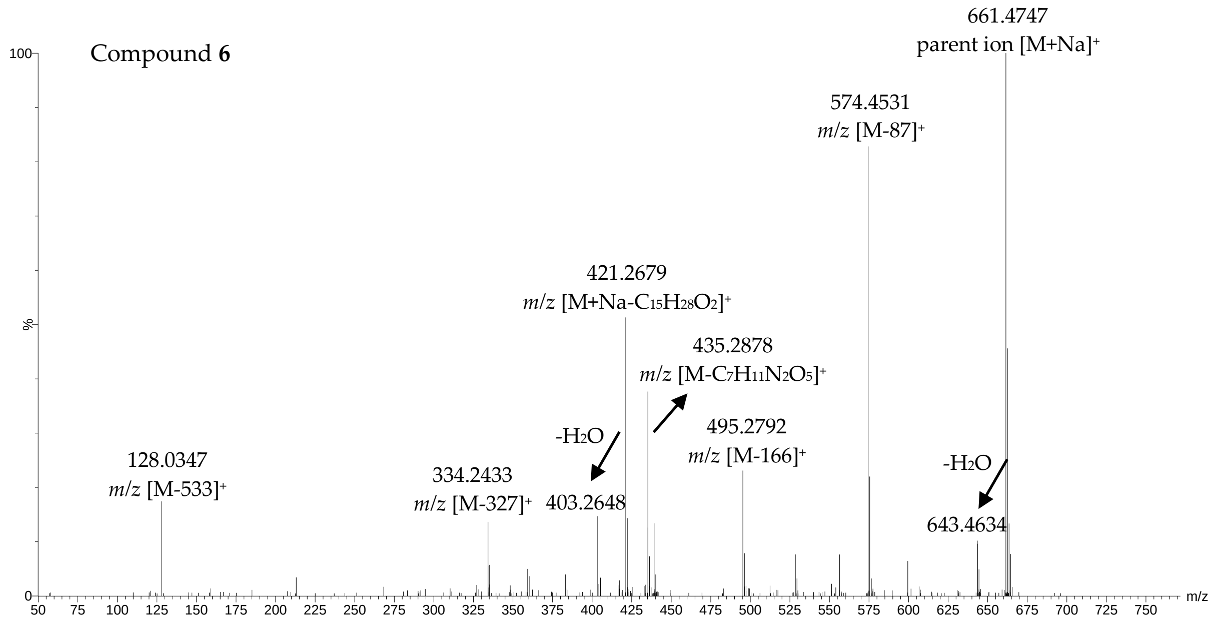

Compound **7**

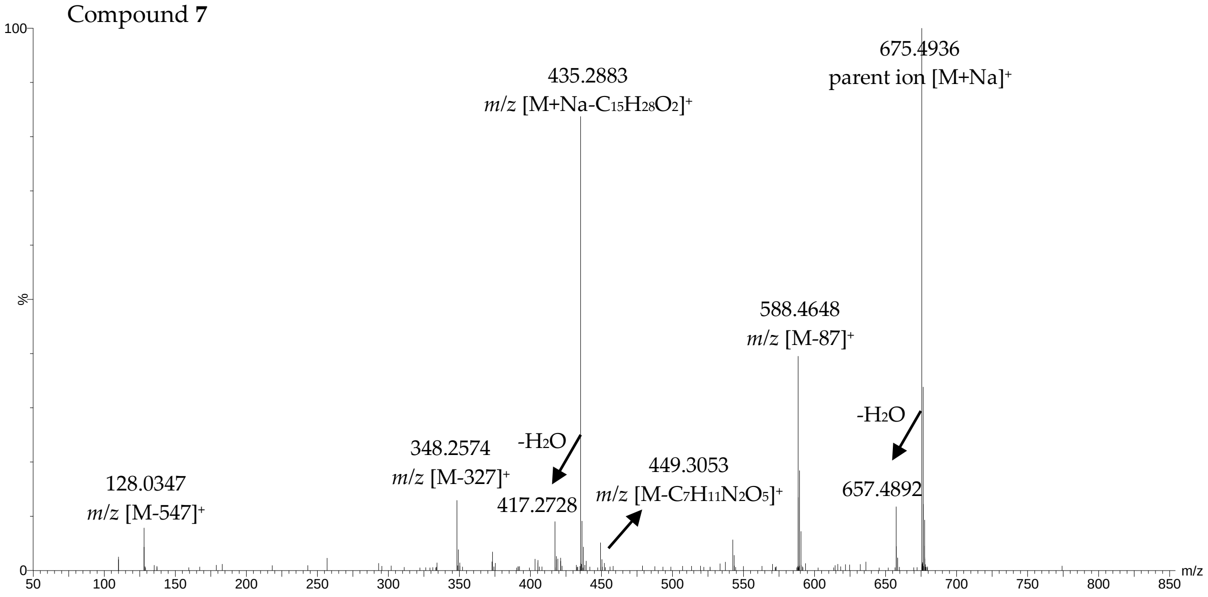
100
%
0
675.4936
parent ion [M+Na]+
435.2883
m/z [M+Na-C15H28O2]+
588.4648
m/z [M-87]+
-H2O
657.4892
-H2O
417.2728
348.2574
m/z [M-327]+
449.3053
m/z [M-C7H11N2O5]+
128.0347
m/z [M-547]+
50 100 150 200 250 300 350 400 450 500 550 600 650 700 750 800 850
m/z
